# Policy relevant results from an expert elicitation on the health risks of phthalates

**DOI:** 10.1186/1476-069X-11-S1-S6

**Published:** 2012-06-28

**Authors:** Karin Elisabeth Zimmer, Arno Christian Gutleb, Solveig Ravnum, Martin Krayer von Krauss, Albertinka J Murk, Erik Ropstad, Janneche Utne Skaare, Gunnar Sundstøl Eriksen, Jan Ludvig Lyche, Janna G Koppe, Brooke L Magnanti, Aileen Yang, Alena Bartonova, Hans Keune

**Affiliations:** 1 Department of Basic Sciences and Aquatic Medicine, Norwegian School of Veterinary Science, Department of Production Animal Clinical Science, P.O.Box 8146, 0033 Oslo, Norway; 2 Department of Environment and Agro-biotechnologies (EVA), Centre de Recherche Public - Gabriel Lippmann, Department of Environment and Agro-biotechnologies (EVA), 41 rue du Brill, 4422 Belveaux, Grand-Duchy of Luxembourg; 3 NILU - Norwegian Institute for Air Research, P.O.Box 100, 2027 Kjeller, Norway; 4 Norwegian Veterinary Institute, P.O.Box 750, 0106 Oslo, Norway; 5 WHO, Regional Office for Europe, Scherfigs vej 8, 2100 Copenhagen Ø, Denmark; 6 Section of Toxicology, Wageningen University, P.O. Box 6700 EA, Wageningen, The Netherlands; 7 Wageningen-IMARES, 1976CP, IJmuiden, The Netherlands; 8 Department of Production Animal Clinical Science, Norwegian School of Veterinary Science, P.O.Box 8146, 0033 Oslo, Norway; 9 Department of Food Safety and Infection Biology, Norwegian School of Veterinary Science, P.O.Box 8146, 0033 Oslo, Norway; 10 EcoBaby Foundation, Hollandstraat 6, 3634 AT Loenersloot, The Netherlands; 11 Biophysics group, University Hospital, St. Michael’s Hospital, Southwell Street, Bristol BS2, 8EJ, UK; 12 Research Institute for Nature and Forest (INBO), Brussels; Centre of Expertise for Environment and Health, Faculty of Political and Social Sciences, University of Antwerp; naXys, Namur Center for Complex Systems, University of Namur, Belgium

## Abstract

**Background:**

The EU 6th Framework Program (FP)-funded Health and Environment Network (HENVINET) aimed to support informed policy making by facilitating the availability of relevant knowledge on different environmental health issues. An approach was developed by which scientific agreement, disagreement, and knowledge gaps could be efficiently identified, and expert advice prepared in a way that is usable for policy makers. There were two aims of the project: 1) to apply the tool to a relevant issue; the potential health impacts of the widely used plasticizers, phthalates, and 2) to evaluate the method and the tool by asking both scientific experts and the target audience, namely policy makers and stakeholders, for their opinions.

**Methods:**

The tool consisted of an expert consultation in several steps on the issue of phthalates in environmental health. A diagram depicting the cause-effect chain, from the production and use of phthalates to potential health impacts, was prepared based on existing reviews. This was used as a basis for an online questionnaire, through which experts in the field were consulted. The results of this first round of consultation laid the foundation for a new questionnaire answered by an expert panel that, subsequently, also discussed approaches and results in a workshop. One major task of the expert panel was to pinpoint priorities from the cause-effect chain according to their impact on the extent of potential health risks and their relevance for reducing uncertainty. The results were condensed into a policy brief that was sent to policy makers and stakeholders for their evaluation.

**Results:**

The experts agreed about the substantial knowledge gaps within the field of phthalates. The top three priorities for further research and policy action were: 1) intrauterine exposure, 2) reproductive toxicology, and 3) exposure from medical devices. Although not all relevant information from the cause-effect chain is known for phthalates, most experts thought that there are enough indications to justify a precautionary approach and to restrict their general use. Although some of the experts expressed some scepticism about such a tool, most felt that important issues were highlighted.

**Conclusions:**

The approach used was an efficient way at summarising priority knowledge gaps as a starting point for health risk assessment of compounds, based on their relevance for the risk assessment outcome. We conclude that this approach is useful for supporting policy makers with state-of-the-art scientific knowledge weighed by experts. The method can assist future evidence-based policy making.

## Background

The Health and Environment Network (HENVINET) was a network funded by EU FP6. The main objective of HENVINET was to establish a long-term co-operation between researchers, policy makers, and other stakeholders in the area of environment and health research and risk assessment. Among the methods used to achieve this goal were, reviewing the available literature, interpreting relevant information for risk assessment, disseminating knowledge on environmental health issues for a wider use by different stakeholders, and for supporting informed policy making. HENVINET consisted of four topic groups, each focusing on one of the four priority diseases related to environmental factors identified by European Environment and Health Action Plan (EHAP 2004-2010), namely, asthma and allergies, cancer, neurodevelopmental disorders, and endocrine disruption.

For endocrine disruption, the initial focus was the potential health impacts caused by the plastic additives, phthalates. As a review paper on phthalates that was written by the topic group failed to convey the most important messages to policy makers from a problem-solving perspective [1], it was decided to explore expert elicitation as a possible tool. This was performed in a similar manner to that conducted by Krayer von Krauss et al. [2], by consulting experts in the topic for advice. Although this method has been criticised, it is still considered one of the best options to support policy making before sufficient scientific data exist [3,4]. The expert elicitation is not intended as a substitute for risk assessment. Rather, it is meant to serve as a rapid assessment tool aimed at highlighting core view-points on key knowledge-related issues for policy making.

Phthalates are widely used, especially as additives in polyvinyl chloride (PVC) products. When incorporated into PVC, phthalates are not chemically bound and are therefore easily released, thereby resulting in animal and human exposure [5]. Different phthalates have different uses according to their molecular weight. The higher molecular weight phthalates, such as di (2-ethylhexyl) phthtalate (DEHP), di-isononyl phthalate (DiNP), and di-isodecyl phthalate (DiDP), are typically used in construction materials and PVC products including clothing (footwear, raincoats), food packaging, children’s products (toys, grip bumpers), and medical devices. Lower molecular weight phthalates are used as solvents in cosmetics, insecticides, and pharmaceuticals, in addition to PVC products [6]. Their widespread use means that human exposure unavoidable.

A number of regulatory actions have already been enacted with respect to phthalates. Most of these concern exposure of infants and children to plastic objects, such as toys, which are frequently mouthed. Phthalates including DEHP, DiNP, DiDP, dibutyl phthalate, butylbenzyl phthalate, and di-n-octyl phthalate are banned in the EU and the United States for use in toys at concentrations above 0.1% by mass of the plasticized material.

This paper reports the development, utilisation, and evaluation of an assessment tool (expert elicitation) that pinpoints priority areas for research and policy action by defining crucial knowledge gaps and the degree of scientific confidence in the current knowledge on the topic of concern. For this first trial of the assessment tool, health-related aspects of phthalates were considered with special focus on (DEHP). Thus, the aim of project was to: 1) assess health-related aspects of phthalates by applying this tool, and 2) evaluate the quality of the tool and its effectiveness in policy decision making.

## Methods

### Cause-effect diagram and questionnaire 1

**Figure 1:**
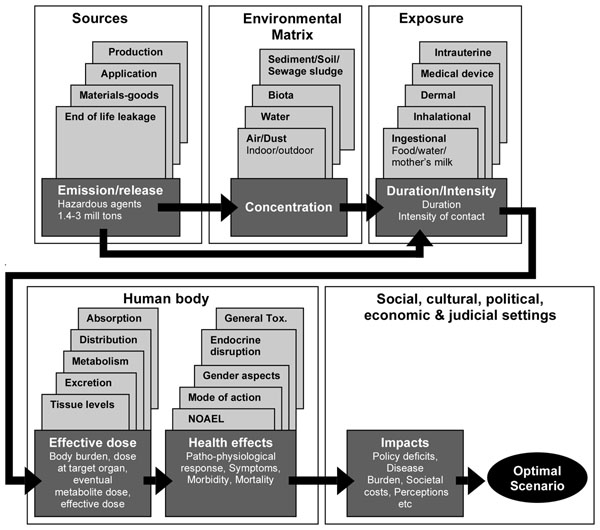
**Phthalate cause-effect chain diagram** Diagram developed by HENVINET to depict the relevant aspects in the cause-effect relationship between sources and production of phthalates and possible health effects. Relevant aspects were identified based on a literature review (Lyche et al., 2009) and updated based on expert comments.

We followed the methods of Keune et al. [7]. Briefly, using the existing review paper [1] as a starting point, a diagram was prepared to illustrate the elements of the cause-effect relationship between production and use of phthalates, and their potential impact on health (Figure 1). Based on this, a questionnaire (Additional file 1, Q1) was developed to evaluate scientists’ confidence in the current knowledge and their opinions on the different issues relating to phthalates as to their relevance for phthalate toxicity. The main aim of this questionnaire was to identify, whether there were areas in the current scientific knowledge in which the experts had generally low confidence and/or there was disagreement regarding the level of confidence. This would assist in identifying issues that are in need of further research and scientific debate. The questionnaire consisted of two parts. Part A was an evaluation of the cause-effect diagram, whilst Part B contained questions on the different elements and sub-elements of the diagram. Based on the scheme of confidence levels used by the Intergovernmental Panel on Climate Change [8], the experts were asked to tick the box representing their confidence levels. These ranged from Very High (VH), High (H), Medium (M), Low (L) to Very Low (VL). The questionnaire was published on the HENVINET web-site for online responses by experts and presented at several international conferences. The online questionnaire (Q1) can be reviewed at the HENVINET website [9] upon logging in, and is also provided in the additional file 1. The questionnaire was created using the Web Content Management Platform, DotNetNuke^® ^[10] add on module called FormMaster. Online forms dedicated to each of the different parts of the questionnaire were prepared. The information from these forms was then saved to a database and later made available to administrators via a custom tool developed to export the database content to an Excel spreadsheet.

Scientists were invited to answer Q1 based on their inclusion in authorships of relevant publications during the past ten years. In addition, information was obtained at relevant conferences and via personal contacts. In the selection, both genders, young and experienced scientists, different areas of expertise (described by the expert in 5 keywords, Table 1), and different nationalities were represented.

**Table 1:** Areas of expert expertise

	No of resp Q1	No of resp Q2
EM: Evironmental chemistry/biomonitoring	4	2
EX: Exposure assessment	2	2
HB: Toxicokinetics, uptake, accumulation, metabolism	3	2
HB: Toxicology, effect studies	12	3
Risk assessment activities	6	2

Self-reported area of expertise for the experts responding to Q1 and Q2. Numbers of experts with expertise in the different fields of phthalates are given for the two different questionnaires. Note that many experts reported expertise in more fields and therefore the sum of respondents in the table is higher than the actual number of experts responding to the questionnaires. EM=Environmental matrix, EX=Exposure, HB=Human Body.

A quantitative assessment of expert consensus in Q1 was performed using the consensus index method [11]. This mathematical measure represents the degree of agreement or disagreement, in which the values are between 0 (perfect disagreement) and 1 (perfect agreement). The consensus is defined as:

Where *d_X_* is the width of *X*, and *d_x_* = *X*_max_ – *X*_min_.

### Questionnaire 2 (Q2) and the workshop

The aims of the 2nd questionnaire (Additional file 3, Q2) were to identify priorities for further policy and research action, and to discuss the implications of the results of the first questionnaire (Additional file 2) for policy and research. The experts were asked to pinpoint priority elements within the cause-effect diagram according to their influence on health risks based on the results from Q1. Questions regarding research needs and justifications of the policy actions were also included.

The selection criterion for experts answering Q2 was expertise in at least one of the main topics of the cause-effect diagram (Figure 1). Six of the experts that responded to Q1, answered Q2. Five of these six experts then met at the workshop in Copenhagen, Denmark, to discuss details of the method and results obtained.

The workshop started by a short review of the results of the Q1 after which the results of the Q2 were discussed. The idea of the expert consultation and the method used were also discussed.

### Evaluation by stakeholders and decision makers

The final report, “*HENVINET policy brief on phthalates”* (Additional file 4), was sent to around 40 policy makers and stakeholders to evaluate the suitability of the method and end-product for the target audience. A short evaluation questionnaire consisting of seven questions was prepared in Microsoft Word (Additional file 5).

## Results

### Cause-effect diagram and questionnaire 1

In total, 15 experts responded to Q1. Due to a technical problem, some answers were lost. As such, there were only five answers to the questions on the importance of the different exposure routes and only 11 answers to questions regarding toxicokinetics.

In Part A of the questionnaire, the evaluation of the cause-effect diagram, about half of the respondents suggested that the diagram did not take into account all the important parameters when evaluating the risks related to production, use, and discharge of phthalates, and that the different diagram elements were not adequately structured. The main critic was that simplicity of such a diagram fails to illustrate important and relevant issues, such as exposure during sensitive stages of life and interactions with other pollutants or chemicals, so called mixed exposures. Where possible, the diagram was changed according to the most relevant comments from the experts (Figure 1).

**Figure 2:**
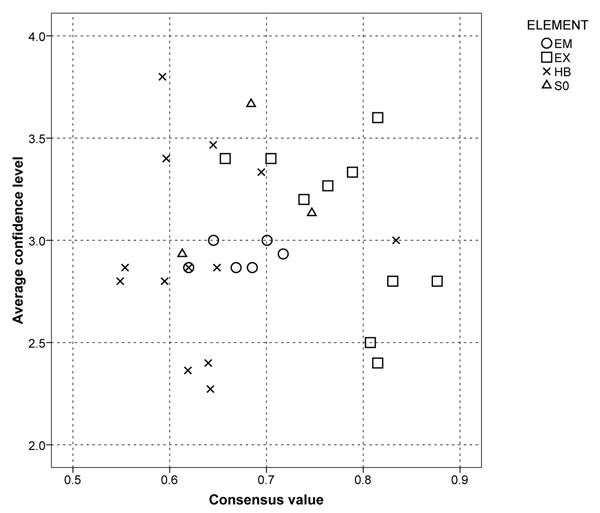
**Results of questionnaire 1** Consensus and average confidence scores of experts in scientific knowledge on all aspects of phthalates: Sources (SO), Environmental Matrix (EM), Exposure (EX), Human Body (HB) of phthalates. The response options ranged from 1 (Very Low) to 5 (Very High) for confidence in the completeness of the existing scientific knowledge. N= 15 (for two questions 11 and for six questions 5).

The full results of Part B of Q1, evaluation of individual model parameters, are provided in additional file 2, while the questions with the lowest and highest confidence and consensus levels are shown in table 2. In general, the consensus among experts differed for the different parts of the questionnaire representing cause-effect diagram elements. For example, the consensus was generally higher for “Exposure” than for “Human body” and these elements also dominated both ends of the confidence scale. The lowest average confidence level was on toxicokinetic issues. The average confidence scores for environmental matrix were all centred around the medium confidence level (3) (Figure 2). The questions with the highest or lowest average consensus, and highest and lowest average confidence scores, are shown in tables 3 a and b, respectively.

**Table 2:** Results of questionnaire 1

a) The questions with consensus (CNS) outside the 10-90 percentile range.										
**Question**	**VL**	**L**	**M**	**H**	**VH**	**No. Resp**	**Mean**	**STD**	**CNS**	**Rank (CNS)**

**Exposure**	1	2	3	4	5					

Levels of exposure in the general population	0	4	10	1	0	15	2,80	0,56	0,83	3
Levels of oral exposure, general population	0	1	4	0	0	5	2,80	0,45	0,88	1
**Human body**										
Adverse health effects in humans	0	3	9	3	0	15	3,00	0,65	0,83	2
Adverse health effects in male experimental animals	2	5	3	4	1	15	2,80	1,21	0,55	32
Mechanisms of action of phtalates	1	2	0	8	4	15	3,80	1,21	0,59	30
Mechanisms of action of phtalate metabolites	3	2	5	4	1	15	2,87	1,25	0,55	31

b) The questions with an average confidence score (Mean) outside the 10-90 percentile range										

**Questions**	**VL**	**L**	**M**	**H**	**VH**	**No. Resp**	**Mean**	**STD**	**CNS**	**Rank (CNS)**

**Source**	1	2	3	4	5					

Annual production volumes of phthalates	0	1	6	5	3	15	3,67	0,90	0,68	16
**Exposure**										
Levels of oral exposure, highly exposed groups	0	0	2	3	0	5	3,60	0,55	0,82	4
Levels of dermal exposure, general population	0	3	2	0	0	5	2,40	0,55	0,82	5
**Human body**										
Final concentration in target tissues	3	3	4	1	0	11	2,27	1,01	0,64	22
Differences in toxicokinetics, identifying sensitive groups	2	5	3	0	1	11	2,36	1,12	0,62	26
Mechanisms of action of phthalates	1	2	0	8	4	15	3,80	1,21	0,59	30
Ability to cause endocrine disruption in the metabolic system	4	2	8	1	0	15	2,40	0,99	0,64	23

Questions with high and low consensus (a) and high and low average score for confidence in the current scientific knowledge (b). The questions were asked: “What is your level of confidence in the currently available data on..” or “What is your level of confidence in our ability to predict…” followed by the question text in the tables. Number of respondents per confidence levels: Very Low (VL) to Very High (VH), total number of respondents, arithmetic mean of confidence score, standard deviation (STD), consensus (CNS) and consensus rank are given. Consensus was measured according to the consensus index method [11].

**Table 3:** Priority areas for further research and policy actions as indicated by the experts.

a) The frequency of the main elements (cause effect chain “groups”).					
*Causal box*	Source	Envir. matrix	**Exposure**	**Human body**	Social

*Frequency*	1	1	**13**	**13**	4

b) The three highest ranked (combination of most frequently mentioned and ranking positions) sub-items within each cause-effect chain element, their frequency and rank and their ranking in total.					

		*Frequency-item*	*Frequency-group*	*Ranking-positions*	*Ranking-total*

**Exposure**			**13**		

Med. device		2		1-3	3

Intrauterine		3		1-2-2	1

**Human body**			**13**		

Reprod. Tox.		3		1-4-4	2

The elements of the cause-effect chain as illustrated by the diagram in Fig 1 were listed according to their influence on the extent of the health risk.

### Questionnaire 2 and workshop discussions

The first question in Q2 asked the evaluators to indicate the most important elements in the cause-effect diagram that will determine the health risk of the phthalates. Different experts interpreted this question differently; some pinpointed sub-elements of the different diagram boxes, while others chose the diagram boxes or elements (the different boxes in Figure 1) as reference points for their answers. The top three priority causal elements of the diagram were identified as 1) intrauterine exposure, 2) reproductive toxicity, and 3) exposure from medical devices (Table 4). There was also a general opinion that mixture effects of phthalates, either mixtures of different phthalates or mixtures of phthalates with other compounds, should be prioritised in research, because this is the real-life scenario. In addition, it was noted that replacements for phthalates should be studied more thoroughly before they are applied on a large scale. These issues were not available as choices from the diagram, but were added as important issues by the experts.

The reason for prioritizing intrauterine exposure was the vulnerability of the foetus to hormone disruption by compounds, most likely involving phthalates. Sensitive windows during foetal development are still poorly characterised, making the relevance high and the confidence low. Several factors, namely, the decreasing sperm counts and quality, and the increasing congenital abnormalities in boys have a suspected environmental cause, and the significant reproductive effects observed in experimental animals exposed to phthalates, meant reproductive toxicology was prioritised. The main reason for exposure from medical devices being indicated as a priority was its considerable contribution to exposing of neonates to phthalates. Furthermore, according to the experts, the lack of knowledge on the two priority issues, intrauterine exposure and reproductive toxicity, justifies obtaining more and better data to improved understanding. In the meantime, policy actions such as monitoring, awareness raising, and restricting activities were suggested for both issues. Also special political actions were advised to protect groups at high risk of intrauterine exposure. According to the experts, suitable substitutes need to be identified in order to reduce the threat of toxic effects on reproduction. For medical devices, policy actions such as awareness raising and restricting and prohibiting activities were the main actions of choice. There was agreement among all experts that enough indications are available to justify such actions, because replacements exist and because this type of exposure is easily reduced. All six experts answering Q2 had medium to very high confidence that conducting more scientific research will yield the decisive knowledge needed on the health risks of phthalates within five years. The confidence spread of the six experts regarding that policy actions to manage the health risks of phthalates will become technically feasible within the next five years, ranged from Low to Very High. The last question addressed the extent to which the current knowledge on health risks of phthalates justifies policy actions. Five out of six experts thought that the general knowledge is sufficient to justify policy actions, such as restricting or prohibiting some compounds. The arguments against restricting actions were the lack of epidemiological studies to prove effects of actual real-life exposure in humans and that there may not be appropriate alternatives for certain products in which case, DEHP should be replaced with less toxic phthalates. The arguments for restricting policies were the observed effects on very sensitive endpoints in experimental animals, and that this justifies the use of the precautionary principle. Proof of human effects is very difficult to achieve because the damage may be made during very limited, sensitive time frames of development.

The expert elicitation and the approach used were discussed at the workshop. Some experts raised the question as to whether such a method is appropriate for producing policy advice and suggested that information on the aim of the project could have been more clearly described. The same experts felt that they were participating in a risk assessment project without being able to update themselves on the necessary literature. Furthermore, they thought that such an elicitation cannot replace the traditional risk assessment because a thorough review of the relevant literature is needed as a basis for policy decisions. It was clarified that the elicitation was intended as a complement to rather than a replacement for the risk assessment processes.

The experts raised several other concerns over the first questionnaire. For instance, the discontinuous scale used from Very Low to Very High is relative and experts found it difficult to commit answers without being allowed to add further explanation. None of the workshop participants felt that they had sufficient competence in all the different parts of the evaluation. Most experts considered that an “outside my area of expertise” option should have been available in the first questionnaire. The background information to the questions of Q1 was criticized for being too condensed and brief. However, experts also thought that the most important issues were pinpointed and that interesting questions had been raised, something that could make a good starting point for risk assessment. During the course of the exercise, it became clearer to some experts why the questions were formulated in the way that they were.

### Evaluation by stakeholders and decision makers

Only three decision maker responses were received. The responses indicated that the policy brief could be of use as a basis for decision-making. However, it was mentioned that more in-depth analyses were needed and that the information provided by the brief is available elsewhere. One policy maker felt that the brief was mostly meant for research policy. Although the content was considered to be clearly presented in the briefs, the policy makers suggested a discussion on restrictions other than a ban on the production and use of phthalates.

## Discussion

A two-step consultation exercise was used to obtain opinions from a group of experts, in order to provide advice to policy makers on areas for research priority and policy measures for problematic compounds. This paper describes not only the results of consultations on the issue of human health risks due to phthalate exposure, but also provides an evaluation of the tool.

The main outcomes of this project, as presented in the policy brief, were those of Q2 and the workshop discussions. The workshop participants agreed that substantial knowledge gaps exist regarding several issues that were considered relevant for human health risks. In particular, additional research is warranted on the levels of exposure and the subsequent health effects, especially on *in utero* exposure and reproductive toxicology. Furthermore, the experts agreed that phthalates should be banned from use in any medical devices, and that research should be initiated that focuses on both alternative substances and the toxic effects of mixtures of phthalates.

### Questionnaire 1

Many questions for which the experts indicated high or low confidence levels also had consensus levels at either extreme of the scale. Interestingly, the consensus levels in human body-related questions were relatively low, although several experts with experience in toxicology were consulted. A low consensus may mean that different experts interpret the available data differently, or that the different experts require different quantities or qualities of evidence in order to feel confident. In addition to areas of low consensus in general, areas with low confidence levels, either with a high, medium, or low consensus among experts, deserve attention in research and policy decisions in order to improve protection of the population from potentially adverse effects of phthalates. The higher consensus levels on questions related to exposure compared with toxicokinetics and toxicology is not surprising since the dose, exposure route, animal model, vast number of endpoints, and methods for extrapolation to humans vary greatly. However, the research conducted during the last decade indicates many uncertainty factors in methods of exposure assessment, both when estimating exposure using data on environmental analyses and behaviours, and when the assessment is based on biomonitoring data [12]. This may be the reason for the relatively high agreement on low confidence in data on oral, inhalational, and dermal exposure. It is worth noting that for the questions regarding these issues, technical problems led to a low number of responses (4-5). According to the Scientific Panel on Food Additives, Flavourings, Processing Aids, and Materials in Contact with Food (AFC), exposure data is scarce and limited to exposure via food, although other sources may contribute significantly [13].

### Questionnaire 2

As indicated by Q1, Human body and Exposure was again ranked as the top priority elements in Q2 for its health risk relevance. Comparing the Q2 priority areas with the results of Q1 is difficult for intrauterine exposure and exposure from medical devices because there were no direct questions on the confidence in these areas in Q1. Reproductive toxicology caused by endocrine disruption had a very low consensus and a medium average score for confidence. The types of action required in the priority fields, according to the experts, also tell something about the confidence in the current knowledge. The experts generally considered more research on intrauterine exposure and reproductive toxicology relevant. This indicates knowledge gaps that should be prioritized in research for better supporting appropriate policy decisions. On the other hand, the strong evidence that medical devices are a considerable source of phthalates for people undergoing surgery or intensive medical care leads to a higher confidence, and therefore stricter regulation, such as a ban, is warranted. The three priority elements, intrauterine exposure, reproductive toxicity, and exposure from medical devices, are connected to each other, suggesting resources should be channelled to research related to reproductive effects caused by early life exposure, and at the same time, policy actions to avoid such exposures should be conducted. This is in accordance with recent literature, stating that there is a pressing need for assessing exposure during critical periods of development, that the levels in medically exposed premature infants are 50 times higher than in children from the general population, and that most developmental toxicity seen in phthalate exposed rodents is connected to the reproductive system [14-16]. The suggestions of the expert panel in the current study to conduct more well-designed, follow-up studies on reproductive systems and development in highly exposed and vulnerable populations is also supported by recent reports [15,17]. However, due to the absence of current clinical or epidemiological evidence they conclude that more research is needed to confirm or reject the suggestions of adverse effects in human infants. Whilst some suggest the use of phthalate-free substitutes in hospitals [15], others state that inadequate health risk assessment of potential alternative substances limits the possibility of replacing DEHP in medical devices [16].

Interestingly, even if the expert opinion was that most of today’s knowledge warrants further research, most experts have both medium to high confidence in that, decisive knowledge will be available, and that policy actions to effectively manage the health risks will become technically feasible, within five years. Even more interesting is the fact that even if more research is needed for most aspects of phthalate health risks, most experts believe the current scientific knowledge is sufficient for using the precautionary principle to take policy actions such as restrictions and banning.

### Methodological considerations

Traditional toxicological risk assessments are resource demanding, they take an extended period of time to conduct, and are based on an overwhelming and ever increasing amount of literature. This, together with the fact that all areas of a chemical are thoroughly covered, makes it easy to lose focus and generate details that are not the most important for the regulatory purposes. Some aspects of a chemical may be more important for regulating its use than others. The described tool of expert elicitation provides a rapid and easy method of highlighting these areas as support for policy making before a full risk assessment is completed and to serve as a starting point for risk assessment. It could therefore be a useful tool for food safety authorities and other risk assessors and decision makers. A few expert elicitations have already been conducted for different environmental health issues [2,18-20]. The present elicitation is unique in its structure in that it is identifying both uncertainty and priorities for policy making, and suggests actions needed to be taken to improve policy making in the field. Also, the elicitation covered all aspects of the case study and the questions asked were quite detailed, also at the level of the cause-effect diagram sub-elements.

Q1 was criticized by the experts mainly because the questions were difficult to interpret and because it did not allow the respondents to explain their answers or skip questions outside their area of expertise. Still, it seemed to have had a necessary role both as a basis for setting priorities, as was the task of Q2, and in preparing the experts for the workshop discussions. The fact that the respondents were not experts in all fields of the subject and still had to answer all questions in Q1 is likely to have influenced the results. Alternatively, the experts could have answered only the questions belonging to their area of expertise as an “outside my area of expertise button” could have been included, or only people with a very broad expertise could have been invited to participate. The first two options would require a substantially increased total number of experts divided equally on the different areas of the field. Another option is that the experts can indicate that a subject is outside of their “core-area of expertise”, after which their response for that subject is weighed according to their self-reported expertise. The experts chosen all had good experience in the field and it was intended that they would answer according to their scientific “gut feeling” rather than using a lot of time to get updated on the latest literature. The experts could have been asked to provide written motivations for their answers as suggested by Knol et al. [3]. This was done for Q2 where the number of experts and questions were limited. For Q1, this could have led to interpretation problems and difficulties in the quantitative analysis of the results. Q1 was first and foremost meant as a preparation for later exercises and, as such, the policy brief mostly deals with the results of Q2 and workshop discussions.

The number of experts attending the workshop was lower than planned and than recommended by Knol et al. [3]. Still, all the major areas of the field were covered by the competence of those present. The task of filling in the questionnaires was challenging for many experts, probably because of the new way of thinking and because some felt that they were participating in a risk assessment procedure without being prepared. Future elicitations should include a more thorough description of the aim of the project, and of how the results can be used.

### Stakeholder evaluation

Only a few evaluation forms were returned from decision makers, therefore, extrapolation from these three responses to the general stakeholder opinion should be done with care. It could also be speculated that the few responses indicated a lack of interest in the tool. Unfortunately, the time the stakeholders and decision makers were given to respond may have been too short and there were also indications that many were very busy during the relevant weeks. An extensive e-mail and the fact that many people refuse to respond to different types of queries may have also contributed to the reduced interest.

## Conclusions and recommendations

An important advantage of the chosen approach is that the experts cannot spend a lot of time obtaining details that may not be relevant for the outcome.

When the background of the group of experts is balanced and covers all aspects of the cause-effect diagram, the chosen approach is suitable to obtain a state-of-the-art expert advice aimed at policy makers about necessary actions.

When applied for phthalates, the main expert advice is to focus research and policy actions on three priority areas: 1) phthalate exposure *in utero*, 2) reproductive toxicology, and 3) exposure to phthalates through medical devices. While there are substantial knowledge gaps in the two former priority areas, there is evidence that exposure from medical devices is substantial and, as alternative compounds exist, this warrants, according to the experts, prohibition of phthalates in such products. Other important areas that should be prioritized are the potential toxic effects of mixtures of phthalates and of alternative compounds. Finally, even though substantial knowledge gaps exist, most experts in our panel think the overall current knowledge legitimizes policy actions that will strongly reduce phthalates from our daily lives.

Although some issues regarding the method used were criticized by the workshop attendants, the tool allowed us to identify valuable recommendations for policy makers and priorities for further research on phthalates. The approach is meant to complement rather than substitute traditional risk assessment by being a rapid assessment tool aimed at creating a basis for selection of core issues for more in-depth analyses, in addition to highlighting different view-points on key knowledge-related issues required for policy making. The HENVINET used the same method for later evaluating knowledge on health risks posed by other chemicals [21,22]. For future use, the method should be refined according to the experiences gained through this project.

## Competing interests

The authors declare that they have no competing interests.

## Authors' contributions

All authors have critically reviewed this manuscript and approved the final version.

KEZ contributed to questionnaire 1, to the later parts of the project including workshop, summary, report (policy brief), evaluation questionnaire, main responsibility for manuscript preparation. ACG was mainly responsible for the first planning phase and the selection of experts. He was also involved in Q1, the workshop, and the report (policy brief).

SR contributed to questionnaire 1, the later parts of the project including workshop, summary, report (policy brief), and evaluation questionnaire. MKvK, work package 1 leader, was heavily involved in the planning and performance at all stages, main responsibility for the workshop. AJM contributed to the early planning phase and in the later parts of the project, mainly in writing the policy brief and manuscript. ER was involved at all stages of planning, and critically reviewed all written deliverables. JUS has also contributed at all stages of planning and critically reviewed all written deliverables. GSE has also contributed at all stages to the planning and critically reviewed all written deliverables. JLL was first author of the review paper which formed the basis for this project, mainly involved in early planning phases, and contributed to the workshop. JGK has also contributed at all stages of planning and critically reviewed all written deliverables. BLM has contributed at all later stages of the planning, critically reviewed all written deliverables and contributed to the workshop. AY was mainly involved in the later stages; in data analysis and interpretation. She also reviewed all written deliverables critically. AB was the overall project leader, involved at all stages of planning and approval of all written deliverables. HK had a major role in planning and performance at all later stages, major role in the workshop and main responsible for preparing and analysing results of Q2, also a special role in the policy brief and last evaluation questionnaire.

## Supplementary Material

Additional file 1 - Q1 **Evaluation questionnaire - causal chain for phthalates** Questionnaire 1 with explanation and background information. Most experts used the online version, which can be found at http://henvinet.nilu.no/EvaluationofKnowledge/tabid/1333/language/en-US/Default.aspx Click here for file

Additional file 2 - results Q1 **Additional file 2. Results of questionnaire 1** Data table with results from all questions of Q1. Mean, standard deviation, consensus measure and rank consensus are given in the table. Click here for file

Additional file 3 - Q2 **Expert Evaluation for phthalates** Questionnaire 2. Experts were expected to look at results of Q1 (Suppl 2-results Q1) when answering (called annex 1 in the questionnaire). Click here for file

Additional file 4 - policy brief **Expert Elicitation on Health Implications of Phthalates** The policy brief which was based on Q2 and the workshop. This is the policy recommendation and the final product from the project. Click here for file

Additional file 5 - Stakeholder questions **Evaluation questionnaire** This questionnaire was used to evaluate the usefulness for the target audience. It was sent to different policy makers and stakeholders. Click here for file
