# Supplementary material for: Policy relevant results from an expert elicitation on the health risks of phthalates
Source: Environ Health. 2012 Jun 28;11(Suppl 1):S6. doi: 10.1186/1476-069X-11-S1-S6 (PMC3388473; doi:10.1186/1476-069X-11-S1-S6)
Supplement: Additional file 1 - Q1 — Evaluation questionnaire - causal chain for phthalates Questionnaire 1 with explanation and background information. Most experts used the online version, which can be found at http://henvinet.nilu.no/EvaluationofKnowledge/tabid/1333/language/en-US/Default.aspx [file 1476-069X-11-S1-S6-S1.pdf]

HENVINET
HEALTH AND ENVIRONMENT NETWORK

Evaluation questionnaire - causal chain for phthalates

Prelude

In order to be better able to summarize the survey results, we would like to ask you to provide basic information about yourself.

- Name (optional):
- Email address (optional):
- Institutional affiliation (required):
- 5 keywords describing your area of expertise (required):
  1.________ 2._______ 3.______ 4._______ 5.________

Introduction

In the Henvinet project we focus on four types of health conditions, “asthma and allergies”, “cancer”, “endocrine disruption”, and “ neurodevelopmental disorders”, and their associations with environmental exposures. This questionnaire is an *evaluation of the quality of the scientific knowledge* of various aspects of the cause-effect relationship between phthalates and endocrine disrupting effects. With your help we will evaluate the quality of the scientific knowledge of various aspects of the cause-effect relationship between phthalates in the environment and endocrine disruption in humans

The goal is to identify knowledge gaps and areas of disagreement between you and your expert colleagues, as well as areas of agreement. Ultimately, the aim is to discuss the implications of these for policy and research.

The evaluation consists of two separate parts. In part A you will be asked to comment on the completeness and structure of a diagram illustrating our current understanding of the cause effect relationship. In part B you will be asked to express your confidence in scientist’s ability to predict the magnitude of a variety of effects of phthalates in the environment.

Often experts feel uncomfortable answering the questions asked, as they feel that they must respond on the basis of a “gut feeling”, setting aside the scientific basis. The scientific basis is carefully referred to in two different aspects of HENVINET reports:

- The results of a detailed literature review are reported in order to explain and justify the causal diagrams produced;
- Relevant literature is referred to in order to explain the basis for any disagreement and trends observed in the survey results.

The results of your evaluation and the evaluations conducted by other eminent authorities in the field will be discussed at a separate workshop within the Henvinet project consortium and with external experts.

We expect the entire exercise will take you about 10-15 minutes. We appreciate your participation very much and, on behalf of the Henvinet consortium we thank you for your time.

Part A. Evaluation of the structure and completeness of the causal diagram

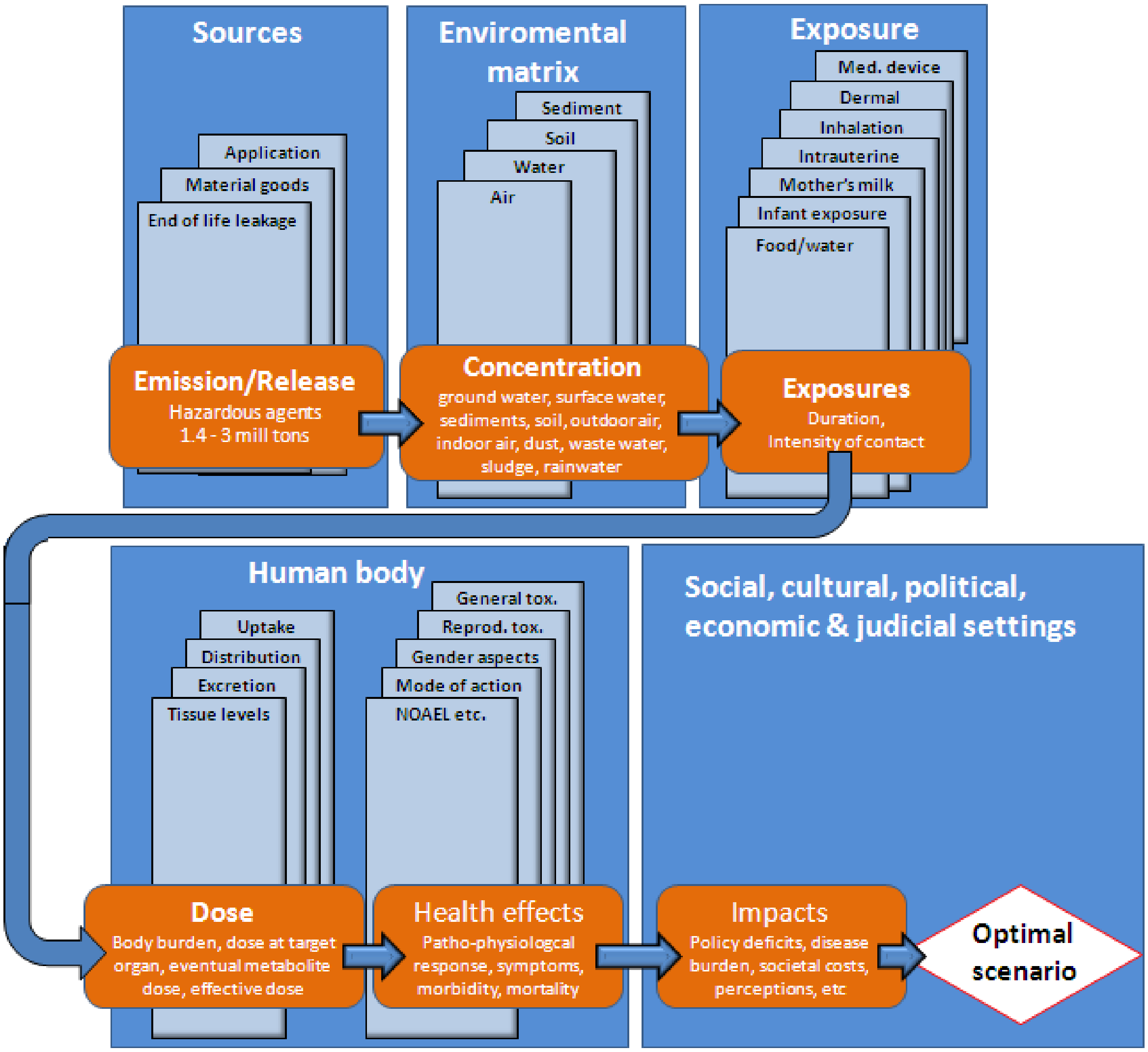

1. Does the diagram take into account all of the important parameters when evaluating the risks related to production, use and discharge of phthalates? **YES/NO**

If No, please explain:

2. Are the different causal relationships adequately structured? **<u>YES/NO</u>**

If No, please explain:

3. Are there any unnecessary parameters shown in the diagram that could be deleted? **YES/NO**

If Yes, please explain:

Part B. Evaluation of individual models or associations

In the questions that follow you will be asked to express your confidence in scientist's ability to predict the concentrations, exposure and effects of phthalates. Insert a check mark where you feel it is appropriate.

It is important that you consider each question **independently** from the others. When answering a question do not take into account the state of knowledge in previous/other questions. As an annex to this questionnaire, you will find summary information related to individual questions, based on HENVINET scientific review to be published soon.

1. Questions about sources

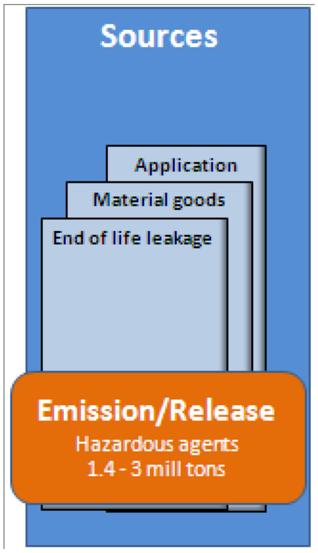

1. What is your level of confidence in our data on the production volumes of phthalates?

(Insert a checkmark in the appropriate box)

4. Very high confidence.<br><br>At least a 9 out of 10 chance of being correct.	3. High confidence.<br><br>At least an 8 out of 10 chance of being correct.	2. Medium confidence.<br><br>At least a 5 out of 10 chance of being correct.	1. Low confidence.<br><br>At least a 2 out of 10 chance of being correct.	0. Very low confidence.<br><br>Less than a 1 out of 10 chance of being correct.

2. What is your level of confidence in our ability to predict the magnitude of emission/release/leakage phthalates during production, transport and use?

(Insert a checkmark in the appropriate box)

4. Very high confidence.<br><br>At least a 9 out of 10 chance of being correct.	3. High confidence.<br><br>At least an 8 out of 10 chance of being correct.	2. Medium confidence.<br><br>At least a 5 out of 10 chance of being correct.	1. Low confidence.<br><br>At least a 2 out of 10 chance of being correct.	0. Very low confidence.<br><br>Less than a 1 out of 10 chance of being correct.

3. What is your level of confidence in our ability to identify and quantify all different applications of phthalates?

(Insert a checkmark in the appropriate box)

4. Very high confidence.<br><br>At least a 9 out of 10 chance of being correct.	3. High confidence.<br><br>At least an 8 out of 10 chance of being correct.	2. Medium confidence.<br><br>At least a 5 out of 10 chance of being correct.	1. Low confidence.<br><br>At least a 2 out of 10 chance of being correct.	0. Very low confidence.<br><br>Less than a 1 out of 10 chance of being correct.

2. Questions about environmental matrices

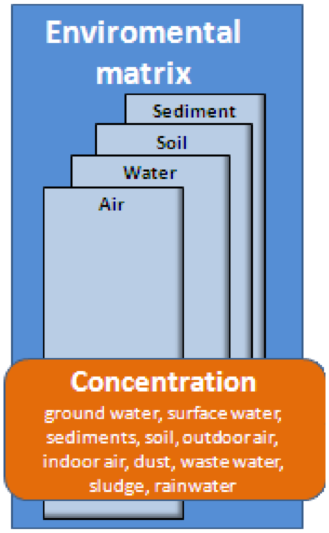

4. What is your level of confidence in our ability to predict the concentration of phthalates in groundwater?

(Insert a checkmark in the appropriate box)

4. Very high confidence.<br>At least a 9 out of 10 chance of being correct.	3. High confidence.<br>At least an 8 out of 10 chance of being correct.	2. Medium confidence.<br>At least a 5 out of 10 chance of being correct.	1. Low confidence.<br>At least a 2 out of 10 chance of being correct.	0. Very low confidence.<br>Less than a 1 out of 10 chance of being correct.

5. What is your level of confidence in our ability to predict the concentration of phthalates in sediments?

(Insert a checkmark in the appropriate box)

4. Very high confidence.<br>At least a 9 out of 10 chance of being correct.	3. High confidence.<br>At least an 8 out of 10 chance of being correct.	2. Medium confidence.<br>At least a 5 out of 10 chance of being correct.	1. Low confidence.<br>At least a 2 out of 10 chance of being correct.	0. Very low confidence.<br>Less than a 1 out of 10 chance of being correct.

6. What is your level of confidence in our ability to predict the concentration of phthalates in soil?

(Insert a checkmark in the appropriate box)

4. Very high confidence.<br><br>At least a 9 out of 10 chance of being correct.	3. High confidence.<br><br>At least an 8 out of 10 chance of being correct.	2. Medium confidence.<br><br>At least a 5 out of 10 chance of being correct.	1. Low confidence.<br><br>At least a 2 out of 10 chance of being correct.	0. Very low confidence.<br><br>Less than a 1 out of 10 chance of being correct.

7. What is your level of confidence in our ability to predict the concentration of phthalates in outdoor air?

(Insert a checkmark in the appropriate box)

4. Very high confidence.<br><br>At least a 9 out of 10 chance of being correct.	3. High confidence.<br><br>At least an 8 out of 10 chance of being correct.	2. Medium confidence.<br><br>At least a 5 out of 10 chance of being correct.	1. Low confidence.<br><br>At least a 2 out of 10 chance of being correct.	0. Very low confidence.<br><br>Less than a 1 out of 10 chance of being correct.

8. What is your level of confidence in our ability to predict the concentration of phthalates in indoor air and dust?

(Insert a checkmark in the appropriate box)

4. Very high confidence.<br><br>At least a 9 out of 10 chance of being correct.	3. High confidence.<br><br>At least an 8 out of 10 chance of being correct.	2. Medium confidence.<br><br>At least a 5 out of 10 chance of being correct.	1. Low confidence.<br><br>At least a 2 out of 10 chance of being correct.	0. Very low confidence.<br><br>Less than a 1 out of 10 chance of being correct.

9. What is your level of confidence in our ability to predict environmental transformation and biological half-lives for phthalates?

(Insert a checkmark in the appropriate box)

4. Very high confidence.<br><br>At least a 9 out of 10 chance of being correct.	3. High confidence.<br><br>At least an 8 out of 10 chance of being correct.	2. Medium confidence.<br><br>At least a 5 out of 10 chance of being correct.	1. Low confidence.<br><br>At least a 2 out of 10 chance of being correct.	0. Very low confidence.<br><br>Less than a 1 out of 10 chance of being correct.

3. Questions related to exposures

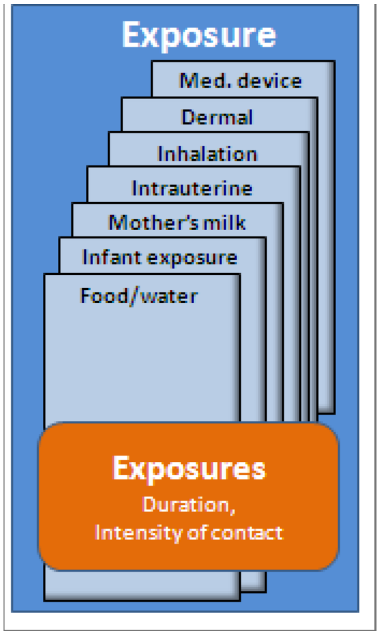

10. What is your level of confidence in our ability to predict the *levels of exposure* to phthalates in the *general* populations?

(Insert a checkmark in the appropriate box)

4. Very high confidence.<br><br>At least a 9 out of 10 chance of being correct.	3. High confidence.<br><br>At least an 8 out of 10 chance of being correct.	2. Medium confidence.<br><br>At least a 5 out of 10 chance of being correct.	1. Low confidence.<br><br>At least a 2 out of 10 chance of being correct.	0. Very low confidence.<br><br>Less than a 1 out of 10 chance of being correct.

11. What is your level of confidence in our ability to predict the *main sources of exposure* to phthalates for the *general* population?

(Insert a checkmark in the appropriate box)

4. Very high confidence.<br><br>At least a 9 out of 10 chance of being correct.	3. High confidence.<br><br>At least an 8 out of 10 chance of being correct.	2. Medium confidence.<br><br>At least a 5 out of 10 chance of being correct.	1. Low confidence.<br><br>At least a 2 out of 10 chance of being correct.	0. Very low confidence.<br><br>Less than a 1 out of 10 chance of being correct.

12. What is your level of confidence in our ability to identify and predict the *levels of exposure* to phthalates in highly exposed *groups* in the population?

(Insert a checkmark in the appropriate box)

4. Very high confidence.<br><br>At least a 9 out of 10 chance of being correct.	3. High confidence.<br><br>At least an 8 out of 10 chance of being correct.	2. Medium confidence.<br><br>At least a 5 out of 10 chance of being correct.	1. Low confidence.<br><br>At least a 2 out of 10 chance of being correct.	0. Very low confidence.<br><br>Less than a 1 out of 10 chance of being correct.

13. What is your level of confidence in our ability to identify and predict the *main sources of exposure* to phthalates in highly exposed *groups*?

(Insert a checkmark in the appropriate box)

4. Very high confidence.<br><br>At least a 9 out of 10 chance of being correct.	3. High confidence.<br><br>At least an 8 out of 10 chance of being correct.	2. Medium confidence.<br><br>At least a 5 out of 10 chance of being correct.	1. Low confidence.<br><br>At least a 2 out of 10 chance of being correct.	0. Very low confidence.<br><br>Less than a 1 out of 10 chance of being correct.

14. What is your level of confidence in our ability to predict the *levels of oral exposure* to phthalates in the *general population*?

(Insert a checkmark in the appropriate box)

4. Very high confidence.<br><br>At least a 9 out of 10 chance of being correct.	3. High confidence.<br><br>At least an 8 out of 10 chance of being correct.	2. Medium confidence.<br><br>At least a 5 out of 10 chance of being correct.	1. Low confidence.<br><br>At least a 2 out of 10 chance of being correct.	0. Very low confidence.<br><br>Less than a 1 out of 10 chance of being correct.

15. What is your level of confidence in our ability to predict the *levels of oral exposure* to phthalates in highly exposed *groups*?

(Insert a checkmark in the appropriate box)

4. Very high confidence.<br><br>At least a 9 out of 10 chance of being correct.	3. High confidence.<br><br>At least an 8 out of 10 chance of being correct.	2. Medium confidence.<br><br>At least a 5 out of 10 chance of being correct.	1. Low confidence.<br><br>At least a 2 out of 10 chance of being correct.	0. Very low confidence.<br><br>Less than a 1 out of 10 chance of being correct.

16. What is your level of confidence in our ability to predict the *levels of inhalational exposure* to phthalates in the *general population*?

(Insert a checkmark in the appropriate box)

4. Very high confidence.<br><br>At least a 9 out of 10 chance of being correct.	3. High confidence.<br><br>At least an 8 out of 10 chance of being correct.	2. Medium confidence.<br><br>At least a 5 out of 10 chance of being correct.	1. Low confidence.<br><br>At least a 2 out of 10 chance of being correct.	0. Very low confidence.<br><br>Less than a 1 out of 10 chance of being correct.

17. What is your level of confidence in our ability to predict the *levels of inhalational exposure* to phthalates in highly exposed *groups*?

(Insert a checkmark in the appropriate box)

4. Very high confidence.<br><br>At least a 9 out of 10 chance of being correct.	3. High confidence.<br><br>At least an 8 out of 10 chance of being correct.	2. Medium confidence.<br><br>At least a 5 out of 10 chance of being correct.	1. Low confidence.<br><br>At least a 2 out of 10 chance of being correct.	0. Very low confidence.<br><br>Less than a 1 out of 10 chance of being correct.

18. What is your level of confidence in our ability to predict the *levels of dermal exposure* to phthalates in the *general population*?

(Insert a checkmark in the appropriate box)

4. Very high confidence.<br><br>At least a 9 out of 10 chance of being correct.	3. High confidence.<br><br>At least an 8 out of 10 chance of being correct.	2. Medium confidence.<br><br>At least a 5 out of 10 chance of being correct.	1. Low confidence.<br><br>At least a 2 out of 10 chance of being correct.	0. Very low confidence.<br><br>Less than a 1 out of 10 chance of being correct.

19. What is your level of confidence in our ability to predict the *levels of dermal exposure* to phthalates in highly exposed *groups*?

(Insert a checkmark in the appropriate box)

4. Very high confidence.<br><br>At least a 9 out of 10 chance of being correct.	3. High confidence.<br><br>At least an 8 out of 10 chance of being correct.	2. Medium confidence.<br><br>At least a 5 out of 10 chance of being correct.	1. Low confidence.<br><br>At least a 2 out of 10 chance of being correct.	0. Very low confidence.<br><br>Less than a 1 out of 10 chance of being correct.

4. Questions related to toxicokintetics, toxicology and health effects

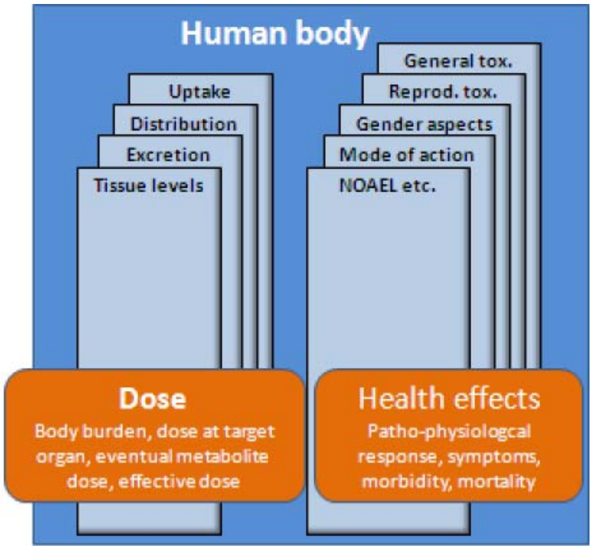

20. What is your level of confidence in our ability to predict the final concentrations in the target tissues, taking factors such as absorption, distribution, metabolism and excretion into consideration?

(Insert a checkmark in the appropriate box)

4. Very high confidence.<br><br>At least a 9 out of 10 chance of being correct.	3. High confidence.<br><br>At least an 8 out of 10 chance of being correct.	2. Medium confidence.<br><br>At least a 5 out of 10 chance of being correct.	1. Low confidence.<br><br>At least a 2 out of 10 chance of being correct.	0. Very low confidence.<br><br>Less than a 1 out of 10 chance of being correct.

21. What is your level of confidence in our ability to predict differences in toxicokinetics, in view of identifying sensitive groups (age, gender etc)?

(Insert a checkmark in the appropriate box)

4. Very high confidence.<br><br>At least a 9 out of 10 chance of being correct.	3. High confidence.<br><br>At least an 8 out of 10 chance of being correct.	2. Medium confidence.<br><br>At least a 5 out of 10 chance of being correct.	1. Low confidence.<br><br>At least a 2 out of 10 chance of being correct.	0. Very low confidence.<br><br>Less than a 1 out of 10 chance of being correct.

22. Based on experimental studies, what is your level of confidence in our ability to predict adverse health effects caused by phthalates in

a) Males?

(Insert a checkmark in the appropriate box)

4. Very high confidence.<br><br>At least a 9 out of 10 chance of being correct.	3. High confidence.<br><br>At least an 8 out of 10 chance of being correct.	2. Medium confidence.<br><br>At least a 5 out of 10 chance of being correct.	1. Low confidence.<br><br>At least a 2 out of 10 chance of being correct.	0. Very low confidence.<br><br>Less than a 1 out of 10 chance of being correct.

b) Females?

(Insert a checkmark in the appropriate box)

4. Very high confidence.<br><br>At least a 9 out of 10 chance of being correct.	3. High confidence.<br><br>At least an 8 out of 10 chance of being correct.	2. Medium confidence.<br><br>At least a 5 out of 10 chance of being correct.	1. Low confidence.<br><br>At least a 2 out of 10 chance of being correct.	0. Very low confidence.<br><br>Less than a 1 out of 10 chance of being correct.

23. What is your level of confidence in our ability to predict adverse health effects in humans caused by environmental exposure to phthalates?

(Insert a checkmark in the appropriate box)

4. Very high confidence.<br><br>At least a 9 out of 10 chance of being correct.	3. High confidence.<br><br>At least an 8 out of 10 chance of being correct.	2. Medium confidence.<br><br>At least a 5 out of 10 chance of being correct.	1. Low confidence.<br><br>At least a 2 out of 10 chance of being correct.	0. Very low confidence.<br><br>Less than a 1 out of 10 chance of being correct.

24. What is your level of confidence in our ability to predict the NOAEL of

a) Single ortho-phthalates?

(Insert a checkmark in the appropriate box)

4. Very high confidence.<br><br>At least a 9 out of 10 chance of being correct.	3. High confidence.<br><br>At least an 8 out of 10 chance of being correct.	2. Medium confidence.<br><br>At least a 5 out of 10 chance of being correct.	1. Low confidence.<br><br>At least a 2 out of 10 chance of being correct.	0. Very low confidence.<br><br>Less than a 1 out of 10 chance of being correct.

b) Mixtures of phthalates?

(Insert a checkmark in the appropriate box)

4. Very high confidence. At least a 9 out of 10 chance of being correct.	3. High confidence. At least an 8 out of 10 chance of being correct.	2. Medium confidence. At least a 5 out of 10 chance of being correct.	1. Low confidence. At least a 2 out of 10 chance of being correct.	0. Very low confidence. Less than a 1 out of 10 chance of being correct.

25. What is your level of confidence in our ability to predict adverse health effects in humans caused by environmental exposure to phthalates?

(Insert a checkmark in the appropriate box)

4. Very high confidence. At least a 9 out of 10 chance of being correct.	3. High confidence. At least an 8 out of 10 chance of being correct.	2. Medium confidence. At least a 5 out of 10 chance of being correct.	1. Low confidence. At least a 2 out of 10 chance of being correct.	0. Very low confidence. Less than a 1 out of 10 chance of being correct.

26. What is your level of confidence in the validity of the claim that only/mainly ortho-phthalates (DEHP, DBP and BBP) have the potential to cause detrimental health effects?

(Insert a checkmark in the appropriate box)

4. Very high confidence. At least a 9 out of 10 chance of being correct.	3. High confidence. At least an 8 out of 10 chance of being correct.	2. Medium confidence. At least a 5 out of 10 chance of being correct.	1. Low confidence. At least a 2 out of 10 chance of being correct.	0. Very low confidence. Less than a 1 out of 10 chance of being correct.

27. What is your level of confidence in the validity of the claim that phthalates and/or their metabolites cause endocrine disrupting effects in

The reproductive system

(Insert a checkmark in the appropriate box)

4. Very high confidence. At least a 9 out of 10 chance of being correct.	3. High confidence. At least an 8 out of 10 chance of being correct.	2. Medium confidence. At least a 5 out of 10 chance of being correct.	1. Low confidence. At least a 2 out of 10 chance of being correct.	0. Very low confidence. Less than a 1 out of 10 chance of being correct.

The thyroid system?

(Insert a checkmark in the appropriate box)

4. Very high confidence. At least a 9 out of 10 chance of being correct.	3. High confidence. At least an 8 out of 10 chance of being correct.	2. Medium confidence. At least a 5 out of 10 chance of being correct.	1. Low confidence. At least a 2 out of 10 chance of being correct.	0. Very low confidence. Less than a 1 out of 10 chance of being correct.

The metabolic system?

(Insert a checkmark in the appropriate box)

4. Very high confidence. At least a 9 out of 10 chance of being correct.	3. High confidence. At least an 8 out of 10 chance of being correct.	2. Medium confidence. At least a 5 out of 10 chance of being correct.	1. Low confidence. At least a 2 out of 10 chance of being correct.	0. Very low confidence. Less than a 1 out of 10 chance of being correct.

5. Overall assessment

28. What is your level of confidence in our ability to predict harmful effects of phthalates in the environment and applications on human health?

? (Insert a checkmark in the appropriate box)

4. Very high confidence. At least a 9 out of 10 chance of being correct.	3. High confidence. At least an 8 out of 10 chance of being correct.	2. Medium confidence. At least a 5 out of 10 chance of being correct.	1. Low confidence. At least a 2 out of 10 chance of being correct.	0. Very low confidence. Less than a 1 out of 10 chance of being correct.

Final comments

The results will now be analysed and discussed by experts within the HENVINET consortium. Are there any comments you would like to make in closing to complete your evaluation? Perhaps you would like to comment on key areas of knowledge which you think are underdeveloped? Perhaps you would like to provide your impressions of the usefulness of this evaluation, or provide suggestions on how to improve it?

Answer:

Thank you very much for taking part in this evaluation. Your contribution is much appreciated.

6. Summary information on individual questions

Sources

What is your level of confidence in our data on the production volumes of phthalates?

The total annual global production of all phthalates is estimated to be 3x10*6 tons, of which 2/3 is DEHP.

What is your level of confidence in our ability to predict the magnitude of emission/release/leakage phthalates during production, transport and use?

Phthalate esters and their metabolites are constantly detected in the indoor environment, consumer products, human urine, mother's milk and amniotic fluid.

Phthalates incorporated in PVC are not covalently bound and are therefore easily released into the environment.

What is your level of confidence in our ability to identify and quantify all different applications of phthalates?

Uses of various phthalates depend on their molecular weight (MW):

- Higher MW phthalates such as DEHP, DiNP and DiDP are used as plasticizers to impart flexibility and durability in polyvinylchloride (PVC) products in construction material, clothing and furnishing.
- Low MW phthalates such as DEP, DMP and DBP are used as solvents in cosmetics, insecticides, pharmaceuticals, construction materials, car products, clothing, food package, children products and medical devices.

Environmental matrix

What is your level of confidence in our ability to predict the concentration of phthalates in groundwater?

- Reported DEHP concentrations in ground water from the United States were reported as mean 15.7µg/l, range nd-470µg/l, while concentrations in Europe were 0.26µg/l with a range from <0.07µg/l to 1.4ug/l. Concentrations for drinking water in Europe ranged from <0.18µg/l to 3.5µg/l. Sample sizes were in the range from 2 to 9 samples investigated.

What is your level of confidence in our ability to predict the concentration of phthalates in sediments?

- DEHP concentration in sediments from Europe were found to be generally low with mean measurable reported concentrations of 4.9µg/g (range: 0.0001-487µg/g; n=405)
- Microorganisms present in the sediments are responsible for the major routes of breakdown of DEHP
- Sediment fingerprints of phthalates showed good correlation with per capita consumption for the high molecular phthalates.

What is your level of confidence in our ability to predict the concentration of phthalates in soil?

- Reported levels for DEHP from European countries were 48 µg/kg mean with a range of 4-5100 (n=3). In the US comparable concentrations ranged from 0.03µg/kg to 1280µg/kg (data points 1)

What is your level of confidence in our ability to predict the concentration of phthalates in outdoor air?

- Concentrations of DEHP measured in Europe show a mean level of 21.9 ng/m^3 and a range of >0.28-1090 ng/m^3 (n=85).
- DEHP concentrations were found to be 1000 times lower in outdoor than in than indoor air in a Japanese study
- Air concentrations were found to be higher in summer than in winter, probably due to enhanced vaporization from plastics.
- Atmospheric transport is important for the presence of phthalates in the Arctic

What is your level of confidence in our ability to predict the concentration of phthalates in indoor air and dust?

- Phthalates and their metabolites are constantly detected in the indoor environment.
- Indoor air DEHP concentrations are found to be up to 1000 times higher than in outdoor air and reached a maximum of 3.13ug/m^3. The air in 27 houses around Tokyo was measured in the study.
- However, studies from Europe showed a mean indoor air concentration for DEHP of 245 ng/m^3 with a range of 18-1046 ng/m^3 (n=398). The levels in dust were found to be within a range of 0.002-4.58 g/kg and have a mean concentration of 0.62 g/kg (n=55).
- A Norwegian study found higher concentrations of DBP in indoor dust of different particle sizes than DEHP. The concentration varied a 10-fold between different sample sites.
- PVC floors are a potential source for phthalates in indoor air; however, PVC-coated wall coverings are found not to release sufficient quantities to lead to intake in the range of the acceptable daily intake (ADI) values.

What is your level of confidence in our ability to predict environmental transformation and biological half-lives for phthalates?

- Phthalates monoesters have a biological half-life of approximately 12hrs.
- Microorganisms present in sediments provide a major route of breakdown of DEHP
- Degradation half-life of DEHP in wastewater is 1.6 days.

Exposures

What is your level of confidence in our ability to predict the *levels of exposure* to phthalates in the *general* populations?

- DEHP exposure was estimated in 2000 to be 3-30 µg/kg body weight / day for adults, 2-3 times higher for children.
- More than 90% of the estimated DEHP intake for adults is from food, whereas formula-fed and breast-fed babies retain only 44% and 60% of the total DEHP from food.
- Reduction of DEHP exposure by 40% from 1996 – 2003 in Germany.
- Median total intake of DEHP range from 8.2µg/kg bw/day in adults up to 25.8µg/kg bw/day in toddlers.
- The secondary DEHP metabolites in urine give a more accurate estimate of the DEHP exposure than the primary monoester.

What is your level of confidence in our ability to predict the *main sources of exposure* to phthalates for the *general* population?

- The main source of exposure for the general population is through ingestion of contaminated food through production and packaging.

What is your level of confidence in our ability to identify and predict the *levels of exposure* to phthalates in highly exposed *groups* in the population?

Children:
Breast milk contained 0.062 ug/g DEHP
Baby food levels were 0.36-0.63 µg/g food.
Infant formula levels were 0.04-0.06 µg/g food.
Infants consuming formula are estimated to be exposed to 8-13 µg/kg bw/day.
Infants fed on breast milk are estimated to be exposed to 8-21 µg/kg bw/day.
It was estimated that a 3 kg child will get 2.5-16.1 µg/kg bw/day, which is well below the European Commission TDI of 37 µg/kg bw/day.
Exposure from toys is estimated to be 1.74 µg/min/10 cm^2.

Medical patients:
Exposure from medical devices is estimated to be:
From parenteral nutrition -> 4-20 mg/day of DEHP is leaching from tubings.
From respiratory therapies-> lower than the detection limits.
From blood transfusions -> > 4 mg/kg bw/day of DEHP (FDA)
From donating and receiving platelets -> 48.1 µg/kg bw
From dialysis -> 59.6 mg during a 4-hour dialysis.
From medication-> exceeded the TDI 4-fold after intake of only four tablets.

Industrial workers:
PVC flooring material increased the air concentrations for the first 150 days after which concentration tended to level off at approximately 1ug/m^3.

Women in reproductive age between 20 and 40:
Exposure can occur from different beauty products (Data from USA).

What is your level of confidence in our ability to identify and predict the *main sources of exposure* to phthalates in highly exposed *groups*?

- Beauty products that contain DBP, such as deodorants, perfumes, hair gels, hair sprays, nail polish and body lotions -> Women in reproductive age between 20 and 40 years using cosmetics are exposed.
- Ingested, inhaled or absorbed phthalates from the mother -> Fetus are exposed through placenta (rodent studies).
- Ingested, inhaled or absorbed phthalates from the mother -> Newborns are exposed through lactation (rodent and human studies).
- Toys and child care articles containing phthalates, mainly DEHP, DBP, BBP and DiDP Four is mentioned (All three are now banned by EU. USA has permitted use of DiNP) -> Children between 0.5 – 4 years of age that mouth, suck or chew on toys are exposed.
- Baby care products, such as lotion, powder and shampoo -> Babies are exposed.
- Factories producing unfoamed PVC flooring -> Industrial workers are exposed.
- Medical devices for administration of medicine and nutrients used during blood transfusions and haemodialysis, may contain very high DEHP levels (20-40%) -> Individuals undergoing medical interventions are exposed.
- Certain pharmaceuticals coated with phthalates, such as antibiotics, antihistamines and laxatives -> Patients taking the drugs are exposed.

What is your level of confidence in our ability to predict the *levels of oral exposure* to phthalates in highly exposed *groups* and the *general population*?

- Oral ingestion of phthalates originates from contaminated food, mothers milk and toys.
- The general dietary intake of DEHP and DBP is estimated to be highest in infants and children between 1-6 years and the exposure is in the range of the tolerable daily intake (TDI) (0.05 mg/kg/day).
- More than 90% of the estimated DEHP intake for adults is from food, whereas formula-fed and breast-fed babies retain only 44% and 60% of the total DEHP from food.
- Studies in rodents show that phthalates are rapidly absorbed from the intestine, and as much as 90% were detected in urine afterwards. Male human studies show that 67% was excreted in the urine.
- Prepacked food increases the levels. In Japan 11.8 ug/g DEHP were detcted in prepacked food.
- Heating of prepacked food in a microwave resulted in 92.2% of the TDI of DEHP.
- Total diets of adults in Denmark in 2000 contained less than 0.188 ug/g DEHP? resulting in a minimum and maximum daily intake of 2.7 and 4.3 ug/kg bw/day.
- A more recent study in Germany resulted in a daily intake of DEHP with a range between 1.0 and 4.2 ug/kg bw/day.
- Exposure from toys is estimated to be 1.74 ug/min/10 cm3.

What is your level of confidence in our ability to predict the *levels of inhalational exposure* to phthalates in highly exposed *groups* and the *general population*?

- Phthalates are absorbed after inhalation in humans.
- Indoor air concentration is 3.13 ug/m^3 (DEHP measured in Japan).
- Exposure from building materials is maximum 3.1 ug/m^3.
- PVC flooring material increased the air concentrations for the first 150 days after which concentration tended to level off at approximately 1 ug/m3.
- DEHP accounted for more than 80% of the phthalate concentration of household dust of 703 mg/kg (median) and 1763 mg/kg (maximum).
- Indoor DEHP concentrations ranged from 156 ng/m^3 to 458 ngn/m^3 in kindergartens.

- DEHP dust concentrations and children with doctor-diagnosed asthma were significantly correlated (Swedish study).

What is your level of confidence in our ability to predict the *levels of dermal exposure* to phthalates in highly exposed *groups* and the *general population*?

- No human *in vivo* dermal absorption studies are available.
- *In vitro* comparisons show that absorption occurs more rapidly through rat skin than human skin.
- In guinea pigs only 3% and 21% was absorbed and excreted after 1 and 7 days, respectively.

Toxicokinetics

What is your level of confidence in our ability to predict the final concentrations in the target tissues, taking factors such as absorption, distribution, metabolism and excretion into consideration?

- Most data are gained from animal studies, human data are scarce.
- There is high variability between species in toxicokinetics.
- No significant accumulation of phthalates in organs and tissues, less than 1% retained.
- Distributed throughout the body with the blood to all tissues.
- Highest concentrations of DEHP have been measured in liver and kidney.
- The orally ingested di-ester phthalates are metabolized into monoesters by non-specific esterases and lipases, and then further by various oxidation and hydroxylation reactions resulting in secondary metabolites.
- Most of the orally ingested phthalates (70%) are excreted in the urine as secondary metabolites in a male human study, and only 13% is excreted as primary monoesters.
- Secondary metabolites are more accurate biomarkers of exposure, compared to the primary monoesters, since the secondary metabolites account for most of the excreted phthalates.

What is your level of confidence in our ability to predict differences in toxicokinetics, in view of identifying sensitive groups (age, gender etc)?

- Exposure studies in humans measuring primary and secondary metabolites suggest age-related differences in metabolism and/or clearance.
- Premature and term infants have reduced renal clearance, due to lack of glucuronidation pathways, which may increase the internal dose of toxic metabolites. Also human neonates have less pancreatic lipases.
- Sex and ethnicity does not matter for toxicokinetics of phthalates.
- High inter-species variability exists in the first step of biotransformation (lipases) of phthalates.

Toxicology/ Health effects

Based on experimental studies, what is your level of confidence in our ability to predict adverse health effects caused by phthalates in

c) Males?
d) Females?

- Effects of exposure are best studied in males; however, a few studies also look at female reproduction.

- Pathological changes in male reproductive organs and lower testosterone levels have been observed when the animal is exposed prenatally (50 mg/kg DBP or 10 mg/kg DEHP). Increased testosterone levels are seen after postnatal exposure (10 mg/kg bw/day from PND 21-120).
- Reduction of prenatal maternal weight gain and number of pups and increase in postnatal mortality has been observed after exposure to high doses (750 and 1500mg/kg bw/day from GD 3-PND 21). Increased nipple sizes in male offspring were seen at all dose levels (375-1500 mg/kg bw/day) and are also seen in other studies, while accessory reproductive organ developmental effects seen at highest doses. No effects seen in female offspring of this study.
- Reduction of Sertoli cell proliferation and increase in multinucleated germ cells and interstitial hyperplasia, depletion of germinal tubule and decreased seminiferous tubule diameter are findings in lower doses (>100 mg/kg single dose, 100-500 mg/kg bw/day) when exposed during development.
- Low doses (14-23 mg/kg bw/day) have caused small reproductive organ sizes in F1 and F2 generations of male rats, without any histological changes or other adverse reproductive effects.
- Effects on male reproductive organs are similar in animals exposed to a single dose and animals exposed to multiple doses during pre- and postnatal development.
- In females, exposure to DEHP before and during puberty (>=500 mg/kg bw/day) increased serum estradiol, advanced onset of puberty and increased ovarian and uterine weight in marmosets, while also lower doses of 2 mg/kg decreased levels of estradiol and led to disturbances of normal ovarian function in adult rats.

What is your level of confidence in our ability to predict adverse health effects in humans caused by environmental exposure to phthalates?

- Few studies have reported a relationship between environmental exposure and human health.
- Animal studies support the hypothesis that there is a relationship between environmental exposure and human health.
- Effects observed in rat studies resemble testicular dysgenesis syndrome in humans
- Levels of phthalates have been negatively associated with sperm parameters and testosterone and LH concentrations.
- In females, higher levels of phthalates have been associated with endometriosis in a few studies.
- In human boys of 2-36 months of age, a negative association between anogenital distance and phthalate metabolites in their mothers' urine has been detected.

What is your level of confidence in our ability to predict the NOAEL of

c) Single ortho-phthalates?
d) Mixtures of phthalates?

- The Scientific Committee on Toxicity, Ecotoxicity and the Environment (CSTTEE) support the suggested new NOAEL of DEHP of 4.8 mg/kg bw/day for testicular and developmental toxicity, which was found to be the most sensitive endpoint (2004).
- The same committee recommended using not only MEHP, but also 5-OH-MEHP and 5-oxo-MEHP for biomonitoring
- Sensitivity in different species seems to differ considerably. E.g. in mamorsets no testicular effects were seen at a dose of 2500 mg/kg bw/day. This is explained by a lower

absorption from marmoset intestines than from rodent intestines. Rodent liver peroxisome proliferation is regarded not relevant for human risk assessment as rodents are highly sensitive to this phenomenon and that the critical effects of DEHP relate to reproduction.

- A few experimental studies in rats published after 2005 suggest a similar mechanism of action for DEHP, BBP, DBP and DiBP on foetal testicular testosterone production and for DiBP, DEHP, DBP and DiNP on foetal testicular testosterone production and testicular histopathology, but this has not been related to other effects

What is your level of confidence in our knowledge of the mechanism(s) of action of

a) Parent phthalate compounds?
b) Phthalate metabolites?

- A study has shown that only unmetabolized phthalates have affinity for steroid receptors, not the absorbed monoesters. This indicates a lack of receptor-mediated effects *in vivo.*
- MEHP has shown to be a more potent testicular toxicant than the parent compound DEHP. MEHP readily crosses the placenta
- Reduced testosterone concentration following DEHP or DBP might be due to reduced expression of genes involved in steroidogenesis. Phthalates also interfere with expression of other genes involved in testicular descent and cell cycle, causing decreased proliferation, eg of Sertoli cells.
- Peroxisome-proliferatior-activated receptors (PPAR), which are involved in metabolism, cell growth and stress responses, may be involved in testicular toxicity following phthalate exposure. PPAR is probably also responsible for reduced levels of aromatase and thus estradiol.

What is your level of confidence in the validity of the claim that only/mainly ortho-phthalates (DEHP, DBP and BBP) have the potential to cause detrimental health effects?

- The general consensus is that only ortho-phthalates with side-chain length of C4-C6 including DEHP, DBP and BBP have potential to disrupt normal development and reproduction.
- Findings have suggested that these phthalates are endocrine disruptors affecting development, reproductive and thyroid hormonal axes and may contribute to the increase in prevalence of metabolic syndrome.
- Few studies with the other phthalates are available.

What is your level of confidence in the validity of the claim that phthalates and/or their metabolites cause endocrine disrupting effects in

a) The reproductive system
b) The thyroid system?
c) The metabolic system?

- Pathological changes in male reproductive organs and lower testosterone levels have been observed when the animal is exposed prenatally (50 mg/kg DBP or 10mg/kg DEHP). Increased testosterone levels are seen after postnatal exposure (10 mg/kg bw/day from PND 21-120).

- Increased nipple sizes in male offspring were seen at all dose levels (375-1500 mg/kg bw/day) and are also seen in other studies, while accessory reproductive organ developmental effects seen at highest doses
- Reduction of Sertoli cell proliferation and increase in multinucleated germ cells and interstitial hyperplasia, depletion of germinal tubule and decreased seminiferous tubule diameter are findings in lower doses (>100 mg/kg single dose, 100-500 mg/kg bw/day) when exposed during development
- Low doses (14-23 mg/kg bw/day) have caused small reproductive organ sizes in F1 and F2 generations of male rats, without any histological changes or other adverse reproductive effects.
- In females, exposure to DEHP before and during puberty (>=500 mg/kg bw/day) increased serum estradiol, advanced onset of puberty and increased ovarian and uterine weight in marmosets, while also lower doses of 2 mg/kg decreased levels of estradiol and led to disturbances of normal ovarian function in adult rats
- Effects observed in rat studies resemble testicular dysgenesis syndrome in humans
- Levels of phthalates have been negatively associated with sperm parameters and testosterone and LH concentrations.
- In females, higher levels of phthalates have been associated with endometriosis in a few studies.
- In human boys of 2-36 months of age, a negative association between anogenital distance and phthalate metabolites in their mothers' urine has been detected.
- Normal thyroid hormone function has been shown to be important for reproductive system development in females and males.
- Thyroid hormone and TSH levels were inversely correlated with urinary MEHP concentration in humans and animal studies also indicate an association between DEHP exposure and thyroid hormones.
- Significant positive correlations between waist circumference and urinary phthalate metabolites are found in American men.
