# Supplementary material for: Policy relevant results from an expert elicitation on the health risks of phthalates
Source: Environ Health. 2012 Jun 28;11(Suppl 1):S6. doi: 10.1186/1476-069X-11-S1-S6 (PMC3388473; doi:10.1186/1476-069X-11-S1-S6)
Supplement: Additional file 2 - results Q1 — Additional file 2. Results of questionnaire 1 Data table with results from all questions of Q1. Mean, standard deviation, consensus measure and rank consensus are given in the table. [file 1476-069X-11-S1-S6-S2.pdf]

Additional file 2

Zimmer et al 2010: Policy relevant Results from an Expert Elicitation on the Health Risks of phthalates

Results of questionnaire 1. The questions were asked: What is your level of confidence in the currently available data on.. or What is your level of confidence in the our ability to predict… followed by the text in the table. Number of respondents on different confidence levels, very low (VL) to very high (VH), total number of respondents, arithmetic mean of confidence score, standard deviation (STD), consensus (CNS) and consensus rank. A Likert scale, which represents different categories, was applied. Consensus was measured according to the consensus index method (Tastle and Wierman 2006).

Questions	VL	L	M	H	VH	No. Resp	Mean	STD	CNS	Rank (CNS)
**Source**	1	2	3	4	5					
Production volumes	0	1	6	5	3	15	3.67	0.90	0.68	16
Emission release leakage during production transport & use	1	5	4	4	1	15	2.93	1.10	0.61	27
Different applications	0	3	8	3	1	15	3.13	0.83	0.75	9
**Environmental matrix**										
Ground water	1	3	8	2	1	15	2.93	0.96	0.72	11
Sediments	2	2	8	2	1	15	2.87	1.06	0.67	17
Soil	2	3	6	3	1	15	2.87	1.13	0.62	24
Outdoor air	1	4	7	2	1	15	2.87	0.99	0.69	15
Indoor air and dust	1	3	7	3	1	15	3.00	1.00	0.70	13
Environmental transformation and biological halflives	1	4	5	4	1	15	3.00	1.07	0.65	20
**Exposure**										
Levels of exposure in the general population	0	4	10	1	0	15	2.80	0.56	0.83	3
Main exposure sources for the general population	0	1	8	6	0	15	3.33	0.62	0.79	7
Levels of exposure in highly exposed population groups	0	2	7	6	0	15	3.27	0.70	0.76	8
Main exposure sources for the general population	0	2	8	2	3	15	3.40	0.99	0.66	18
**Oral exposure**										
General population	0	1	4	0	0	5	2.80	0.45	0.88	1
Highly exposed groups	0	0	2	3	0	5	3.60	0.55	0.82	4
**Inhalational exposure**										
General population	0	2	2	0	0	4	2.50	0.58	0.81	6
Highly exposed groups	0	1	1	3	0	5	3.40	0.89	0.70	12
**Dermal exposure**										
General population	0	3	2	0	0	5	2.40	0.55	0.82	5
Highly exposed groups	0	1	2	2	0	5	3.20	0.84	0.74	10
**Toxicokinetics**										
Final conc. in target tissues	3	3	4	1	0	11	2.27	1.01	0.64	22

Differences in toxicokinetics	2	5	3	0	1	11	2.36	1.12	0.62	26
Adverse health effects in humans	0	3	9	3	0	15	3.00	0.65	0.83	2
Detrimental health effects	1	1	5	8	0	15	3.33	0.90	0.69	14
**Adverse health effects**										
Males	2	5	3	4	1	15	2.80	1.21	0.55	32
Females	0	3	4	6	2	15	3.47	0.99	0.64	21
**NOAEL of**										
Single orthophthalates	1	2	4	6	2	15	3.40	1.12	0.60	28
Mixtures of phthalates	1	5	4	5	0	15	2.87	0.99	0.65	19
**Mechanisms of actions**										
Phtalates and their metabolites	1	2	0	8	4	15	3.80	1.21	0.59	30
Phtalate metabolites	3	2	5	4	1	15	2.87	1.25	0.55	31
**Endocrine disrupting effects in**										
The metabolic system	4	2	8	1	0	15	2.40	0.99	0.64	23
The thyroid system	2	3	6	3	1	15	2.87	1.13	0.62	25
The reproductive system	2	4	5	3	1	15	2.80	1.15	0.59	29
