# Supplementary material for: Policy relevant results from an expert elicitation on the health risks of phthalates
Source: Environ Health. 2012 Jun 28;11(Suppl 1):S6. doi: 10.1186/1476-069X-11-S1-S6 (PMC3388473; doi:10.1186/1476-069X-11-S1-S6)
Supplement: Additional file 3 - Q2 — Expert Evaluation for phthalates Questionnaire 2. Experts were expected to look at results of Q1 (Suppl 2-results Q1) when answering (called annex 1 in the questionnaire). [file 1476-069X-11-S1-S6-S3.pdf]

HENVINET
HEALTH AND ENVIRONMENT NETWORK

Expert evaluation for phthalates

Introduction

Thank you very much for participating in this expert evaluation on phthalates, conducted in the context of the HENVINET project.

With your help we will further interpret the results of the first evaluation of the state of the art in the scientific knowledge of various aspects of the cause-effect relationship between the production and use of **phthalates** and the potential impact on health. You will find the causal diagram on the following page and a summary of the results of the first evaluation following that. The goal of this questionnaire is to identify priorities for further action and to discuss the implications of the results of the evaluation for policy and research. The outcomes of this questionnaire will form the basis for the workshop at the offices of WHO Euro in Copenhagen on May 19th.

In this questionnaire we will first ask you to pinpoint priority elements in the causal diagram. We will then ask you a series of four questions dealing with the implications of the results of the first evaluation on these priority elements. Issues such as research needs and the policy actions justified will be explored.

In the expert workshop on May 19th, a synthesis of the results of the two questionnaires will be presented and discussed in order to arrive at expert advice for EU-policy-makers.

We appreciate your participation very much and, on behalf of the Endocrine Disruption expert group, WHO Euro and the Henvinet consortium, we thank you for your time.

Causal diagram for phthalates

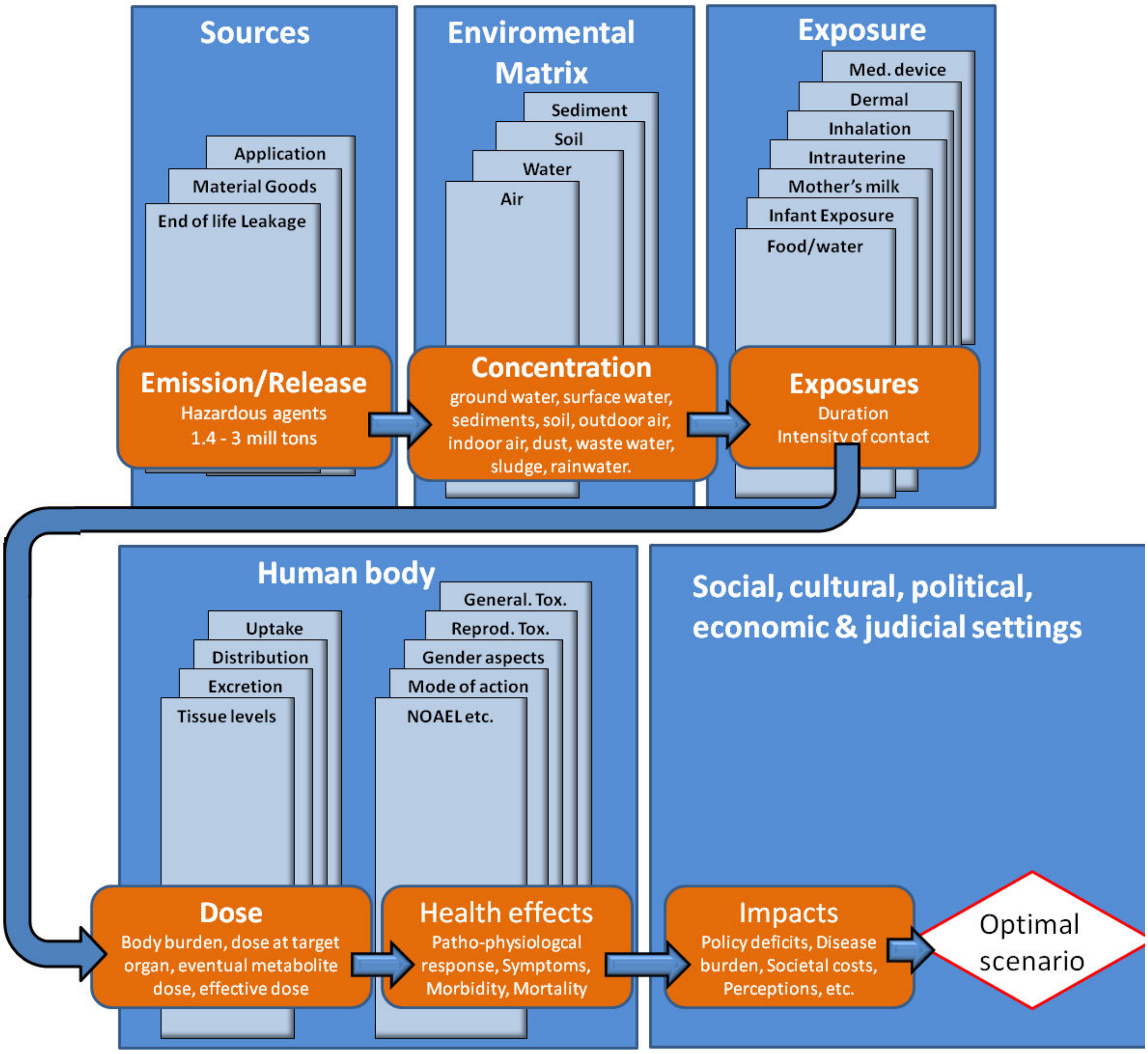

Results of first evaluation questionnaire for phthalates

see attached excel file

Questionnaire for phthalates

1. In the table below, list the five most important elements of the causal diagram for phthalates. Prioritize according to their influence on the extent of the health risk the causal chain leads to. If a small change in the value of an element results in a large change in the health impact, then this element has a high influence and should be considered very important. Conversely, if a large change in the value of an element leads to only small changes in the health impact, then this element has little influence and is not so important.
You may rank no more than two elements equally; five elements in total.

Priority	Label of element	Please explain why you attribute this priority to this element
1.		
2.		
3.		
4.		
5.		

**If you consider elements that are not represented in the causal diagram to be amongst the five most important, you may include these in the priority list. In Annex 1(after the causal diagram) you will find elements suggested by your colleagues as important supplements to the causal diagram we presented.*

2. Different strengths of evidence justify different policy intervention. For example, a high level of evidence is required to justify banning a substance, while a lower level of evidence might be sufficient to justify initiating a targeted monitoring program or a mandatory labelling scheme.

For each of the priority elements you have identified, indicate the type of action you consider is justified by the evidence available.

Causal elements*	*Conduct Scientific research*					*Policy action*<br>Concrete action by policymakers				Please explain the basis for your choice. If you have any specific scientific studies or hypotheses, please specify them here. If there are any broad implications for science, please explain. If you have any specific policy actions in mind, please specify them here.
	Fundamental science to gain knowledge about the problem			*Applied science* to gain knowledge about <u>solving</u> the problem						
	More data	Better data	Better under-standing	Developing interventions	Experimenting with interventions in practice	Monitor-ing	Awareness raising	Restricting risk activities	Prohibiting risk activities	
1.										
2.										
3.										
4.										
5.										

**As ranked by you in question 1*

Phthalates

Before answering the following questions, please take a moment to consider the "big picture" depicted by the overall results of the first evaluation on phthalates (provided in annex 2).

With this in mind:

3. What is your level of confidence that conducting more scientific research would yield <u>decisive</u> knowledge on the risks of phthalates within the next five years? *(decisive knowledge is understood here as knowledge that would clearly dictate which type of policy action is to be undertaken(or not))*

Insert checkmark in appropriate box					Please justify your answer. Also, if you expect decisive knowledge to become available, please specify which of the five causal element(s) you selected in question 1 this knowledge would most likely pertain to.
Very high	High	Medium	Low	Very low	

Interpretive guidance: Very high - At least 9 in 10 chance of being correct; High - At least 7 in 10 chance of being correct; Medium - At least 5 in 10 chance of being correct; Low - At least 3 in 10 chance of being correct; Very low - 2 in 10 or less chance of being correct

4. What is your level of confidence in the possibility that policy actions to effectively manage the health risks of phthalates will become technically (not politically) feasible within the next five years? *In other words, are effective policy actions technically feasible now, or to what extent would you expect them to become feasible within the next 5 years?*

Insert checkmark in appropriate box					Please explain why.
Very high	High	Medium	Low	Very low	

Interpretive guidance: Very high - At least 9 in 10 chance of being correct; High - At least 7 in 10 chance of being correct; Medium - At least 5 in 10 chance of being correct; Low - At least 3 in 10 chance of being correct; Very low - 2 in 10 or less chance of being correct

5. As mentioned above, different strengths of evidence justify different policy intervention.

Considering the overall results of the evaluation, to what extent do you think the current scientific knowledge of the health risks of phthalates represents sufficient evidence to justify policy action (or not)?

Insert checkmark in appropriate box

Insufficient evidence to justify a policy intervention	Sufficient evidence to justify not taking policy action	Sufficient evidence to justify a policy intervention

Please explain why you choose this option.

Thank you !!
