# Supplementary material for: Policy relevant results from an expert elicitation on the health risks of phthalates
Source: Environ Health. 2012 Jun 28;11(Suppl 1):S6. doi: 10.1186/1476-069X-11-S1-S6 (PMC3388473; doi:10.1186/1476-069X-11-S1-S6)
Supplement: Additional file 4 - policy brief — Expert Elicitation on Health Implications of Phthalates The policy brief which was based on Q2 and the workshop. This is the policy recommendation and the final product from the project. [file 1476-069X-11-S1-S6-S4.pdf]

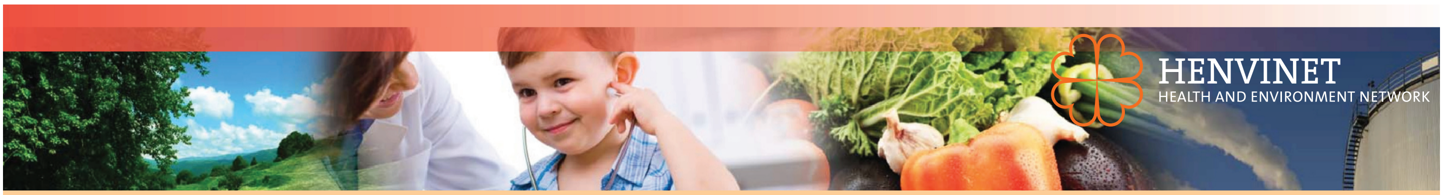

HENVINET Policy Brief:

Expert Elicitation on Health Implications of Phthalates

Arno C Gutleb

Policy context

- Phthalates are widely used in products as additives to PVC products such as food packaging, medical devices, solvents in cosmetics, insecticides and pharmaceuticals or construction materials.
- The major source for the general population is ingestion of food contaminated through production, processing and packaging. Other significant sources are indoor air exposure and cosmetics.
- Persons under intensive care and especially neonates are highly exposed via medical devices.
- Despite uncertainties and differences between various phthalates in respect to the toxicokinetic behaviour the concentrations in children are approximately two fold higher than in adults. Altogether a significant proportion of the population is continuously exposed to these compounds.
- Toxicological effects observed in animal studies include serious effects such as disruption of hormone levels and reproductive toxicity, foetal death, cancer, liver and kidney injuries.
- Phthalates can cross the placenta leading to exposure of the foetus that is followed in early life by exposure via the mother's milk.

Policy options

In order to evaluate the state of the current scientific knowledge and highlight important policy considerations, experts were approached by two questionnaires followed by a workshop (six experts). Based on the answers from the questionnaires and discussion at the workshop, it was concluded that:

- Experts disagree on whether or not the knowledge currently available is sufficient to justify policy action at this point. A majority of experts participating in the workshop feel that while phthalates are not persistent or bioaccumulative the continuous and daily exposure is leading to an exposure scenario that is in its practical effects similar to those with persistent and bioaccumulative compounds. According to this group of experts this is enough to justify a ban for the use in medical devices. One expert felt that more data are required before a decision to change the status quo is justified.
- There is limited knowledge on many aspects of the wide range of different phthalates, but the information available causes concern and speak in favour of more research. More end-user oriented research and monitoring should be funded in order to better understand the health risks.
- The experts selected three priority areas for which more knowledge will support better understanding:
  - The extent of intrauterine exposure in humans in the first trimester of pregnancy.
  - The extent and sources/processes of occupational exposure that will add to the already high oral exposure.
  - Toxicological data on proposed replacement products and the issue of mixture effects.
- More toxicological data should be required from industry. Also, research collaborations between independent institutions could be organised at the European level.
- Effort should also be put on research on potential alternative substances to phthalates.

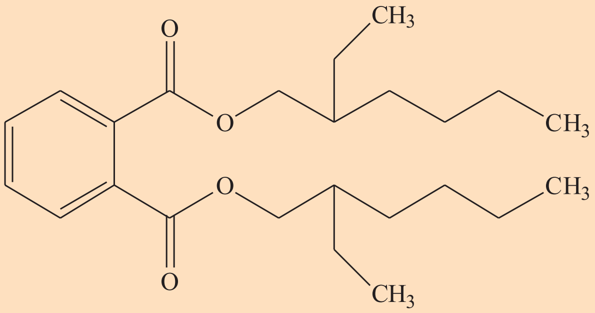

The chemical structure of Bis(2-ethylhexyl)phthalate (DEHP).

Executive summary

Situation

Phthalates are a family of industrial chemicals, which have been used for a variety of purposes such as plasticisers that impart flexibility and durability to polyvinylchloride (PVC) products. They are also used in solvents, lubricating oils, fixatives and as detergents in personal care products. When incorporated into PVC, phthalates are not chemically bound and are therefore easily released into the environment consequently resulting in animal and human exposure (Kavlock et al., 2006).

Annually more than 3 million metric tons of phthalates are used globally, and because of the widespread use, ubiquitous and constant environmental presence exposure of humans, domestic animals and wildlife is virtually unavoidable. Uses of the various phthalates mainly depend on their molecular weight (MW). Higher MW di (2-ethylhexyl) phthalate (DEHP), di-isononyl phthalate (DiNP), and di-isodecyl phthalate (DiDP) are used in construction materials, and numerous PVC products including clothing (footwear, raincoats), food packaging, children's products (toys, grip bumpers), and medical devices. Relatively low MW phthalates such as di-methyl phthalate (DMP), di-ethyl phthalate (DEP), and di-n-butyl phthalate (DBP) tend to be used as solvents and in cosmetics, insecticides and pharmaceuticals, but are also used in PVC (Heudorf et al., 2007).

Background

In the general population the major source of human exposure is through ingestion of food contaminated through production, processing and packaging. Other significant sources are indoor air exposure and possibly via cosmetics. Humans may also be exposed to high doses of phthalates from medical devices during medical procedures such as blood transfusions and hemodialysis. Phthalates and their metabolites were detected in the indoor environment, consumer products, human urine, breast milk, and amniotic fluid (liquid that surrounds and is ingested by the unborn baby). Furthermore, phthalates are also able to cross the placenta, and foetal exposure is closely correlated with maternal exposure (Kavlock et al., 2006; Lyche et al., 2009).

Phthalate esters possess endocrine disrupting properties and exposures to high concentrations were shown to induce foetal death, cancer, malformations, liver and kidney injury and reproductive toxicity in animals (Hauser and Calafat, 2005; Lyche et al., 2009). In humans, particular concerns have been raised regarding adverse effects following exposure to phthalates during development. Phthalates cross from maternal blood into the developing foetus via placental transfer and into neonates via breast milk, and these exposures may affect the developing endocrine system, which is essential for diverse biological functions including, sexual development and reproductive functions in

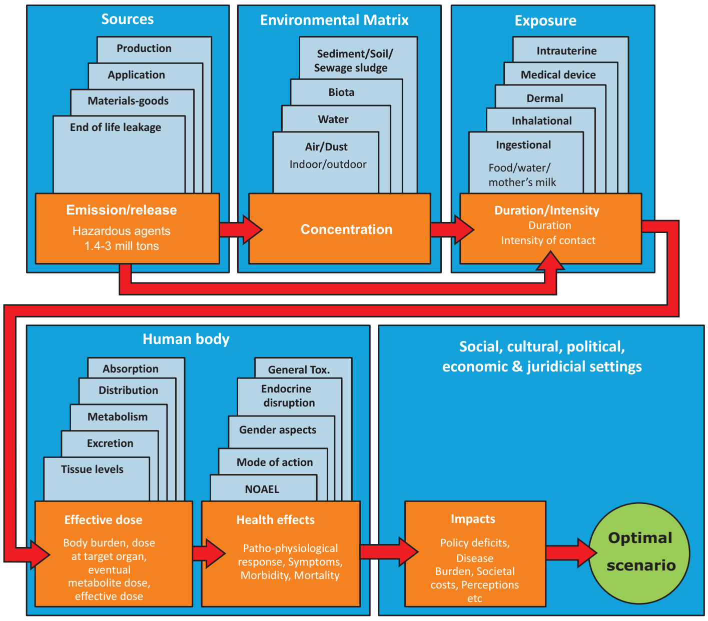

Figure 1. Cause-effect diagram for phthalates based on Lyche et al. (2009). The diagram was used by experts to evaluate the current understanding of the cause-effect relationship between the production and use of phthalates and its potential impact on health. The diagram has been slightly adapted to expert comments.

adults (Kavlock et al., 2006). The adverse effects observed in animals raise concerns as to whether exposure to phthalate esters in the environment represents a potential health risk to humans. The observed high sensitivity of the prenatal developmental stage for endocrine disruption has led to the postulation that increased incidence of human reproductive deficits may be produced by exposure to environmental chemicals during foetal and/or pre-pubertal life (Sharp and Skakkebaek, 2008).

To identify knowledge gaps and potential agreement or disagreement on the different aspects of the phthalates issue a causal diagram illustrating scientists' current understanding of the cause-effect relationship between the production and use of phthalates, especially DEHP and its potential impact in health was made (See Figure 1). The diagram was based on the latest review articles and reports available. A group of experts was asked to express their confidence in the current knowledge in the different parts of the diagram by completing an online questionnaire. From these experts a group of six was selected to complete a second questionnaire and take part in an expert panel workshop where the implications of the results of the two different evaluations for policy and health were discussed. Priorities for further action were identified and the workshop aimed at arriving at a final expert advice for policy makers.

Assessment

In developing an expert advice on phthalates for policy makers an important issue was prioritizing the elements of the

causal diagram with respect to public health risk. This was done in an expert workshop held in Copenhagen in May 2009; six experts participated in this workshop. The ambition was to set priorities for policy uptake.

The priority knowledge gaps

The top area issues that the expert work shop considered to be the most influential for the health impact of phthalates were identified:

- Intensive medical care especially of neonates is known to lead to uptake in patients far exceeding TDIs (Koch et al., 2006; Lyche et al., 2009) and there are already phthalate-free replacement products with identical properties for medical applications available (Pak et al., 2007). There is certainly a need for more research in these areas, also monitoring of levels in humans should be a tool to get a better overview of the exposure situation (Fromme et al., 2007).
- Intrauterine exposure was another important area that should be prioritized as this potentially leads to exposure during critical windows of development leading to life-long health effects (Latini et al., 2006; Mose et al., 2007.
- There is still too little knowledge on potential sources and the extent of occupational exposure in humans that will add to the uptake from food and dust that is already exceeding TDIs in a considerable part of the population (EFSA, 2005; Fromme et al., 2007).
- Mixtures need to be tested as for some phthalates cumulative effects on relevant endpoints such as testosterone production and testicular histopathology have been described (Lyche et al., 2009.

Toxicological health effects were also considered, as an important area to prioritize and pushing the use of alternatives where available. Spreading information on improper use of materials containing phthalates is another area that should get attention (Lyche et al., 2009).

Most experts in the work shop have medium to very high confidence in science coming up with usable or decisive knowledge within the next five years. Experts show medium to high confidence that policy actions to effectively manage the health risks of phthalates are to be technically (not necessarily politically) feasible either now, or will become so within the next five years.

Weight of knowledge

Arguments for using the precautionary principle to ban or restrict the use of phthalates would be the already high proportion of the general population exceeding TDIs combined with the uncertainties and potential threats in the "priority elements" as described above. The effects observed in animal studies involve reproductive development and hormone levels, which are serious effects (Lyche et al., 2009). There is also a risk that other effects appear at lower doses; further research is needed to investigate this. In that case the high environmental concentrations will have even more extensive consequences. Lessons from earlier used persistent compounds should favour precaution also for less persistent compounds where common exposure routes lead to an almost continuous exposure. For some uses, alternative compounds exist, which at least are less likely to leach out of the products they are used for.

On the other hand there are arguments against a ban. The industry may take into use compounds, which are less studied and not toxicologically tested at all. It may also be claimed the existing knowledge does not generate enough understanding to justify a ban, e.g. the current human toxicology data are insufficient to evaluate the prenatal and childhood effects following phthalate exposure.

In the panel of experts, 1 expert was against a ban whereas 5 were in favour of a ban.

Recommendations

Due to the fact that there are substantial gaps in knowledge in both phthalate levels of exposure and consequent health effects in humans, additional research is warranted.

1) It is of key importance to improve the knowledge of human toxicokinetics and toxicity, specifically during pregnancy and the nursing period, because in utero and early postnatal exposure appears to be the most vulnerable period during development.

2) Well-designed follow-up studies of reproductive system development and functions in the most heavily exposed and most vulnerable human populations may address the question of whether phthalates produce adverse human reproductive effects. Reproductive developmental toxicity is well studied in male animals. However, data on female reproductive toxicity are scarce and need further research. Further *in vitro* and *in vivo* studies are also warranted to improve the understanding of the modes of action of phthalates in humans.

3) Most studies focused on adverse reproductive and developmental effects associated with exposure to single phthalates. However, because humans are exposed to mixtures of phthalates both concurrently and sequentially, and available experimental evidence suggests that mixtures of phthalates may induce endocrine disruption in a cumulative fashion, it is necessary to initiate studies, which focus on mixture effects.

4) Phthalates should not be used in any medical device.

5) Despite the need for more knowledge on key issues regarding phthalates, most experts in our panel think that the weight of current knowledge legitimizes policy actions that will strongly reduce phthalates in our daily lives.

Literature

EFSA. 2005. Opinion of the Scientific Panel on Food Additives, Flavourings, Processing Aids and Materials in Contact with Food (AFC) on a request from the Commission related to bis(2-ethylhexyl)phthalate (DEHP) for use in food contact materials. EFSA J. 243:1–20.

Fromme, H., Gruber, L., Schlummer, M., Wolz, G., Böhmer, S., Angerer, J., Mayer, R., Liebl, B., and Bolte, G. 2007. Intake of phthalates and di(2-ethylhexyl)adipate: Results of the integrated exposure assessment survey based on duplicate diet samples and biomonitoring data. Environ. Int. 33:1012–1020.

Hauser, R., and Calafat, A.M. 2005. Phthalates and human health. Occup. Environ. Med. 62:806–818.

Heudorf, U., Mersch-Sundermann, V., and Angerer, J. 2007. Phthalates: Toxicology and exposure. Int. J. Hyg. Environ. Health 210:623–634.

Kavlock, R., Barr, D., Boekelheide, K., Breslin, W., Breysse, P., Chapin, R., Gaido, K., Hodgson, E., Marcus, M., Shea, K., and Williams, P. 2006. NTP-CERHR Expert Panel update on the reproductive and developmental toxicity of di(2-ethylhexyl) phthalate. Reprod. Toxicol. 22:291–399.

Koch, H. M., Preuss, R., and Angerer, J. 2006. Di(2-ethylhexyl)phthalate (DEHP): Human metabolism and internal exposure—An update and latest results. Int. J. Androl. 29:155-165.

Latini, G., Del Vecchio, A., Massaro, M., Verrotti, A., and De Felice, C. 2006. In utero exposure to phthalates and fetal development. Curr. Med. Chem. 13:2527–2534.

Lyche, J.L., Gutleb, A.C., Bergman, Å., Eriksen, G.S., Murk, A.J., Ropstad, E., Saunders, M., Skaare, J.U. 2009. Reproductive and developmental toxicity of phthalates – a review. J. Toxicol. Environ. Health B, Critical Reviews 12, 225-249.

Mose, T., Knudsen, L. E., Hedegaard, M., and Mortensen, G. K. 2007. Transplacental transfer of monomethyl phthalate and mono(2-ethylhexyl) phthalate in a human placenta perfusion system. Int. J. Toxicol. 26:221–229.

Pak, V. M., Nailon, R. E., and McCauley, L. A. 2007. Neonatal exposure to plasticizers in the NICU. MCN Am. J. Matern. Child. Nurs. 32:244–249.

Sharpe, R. M., and Skakkebaek, N. E. 2008. Testicular dysgenesis syndrome: Mechanistic insights and potential new downstream effects. Fertil. Steril. 89(2 suppl.):e33–e38.

Acknowledgements:

All experts attending the workshop are gratefully acknowledged in alphabetical order: Helen Håkansson[a], Janna G Koppe[b], Gavin ten Tuscher[b] and 3 experts who preferred anonymity. Also, all experts responding to the first questionnaire are acknowledged for their valuable and essential contribution.

[a] *Karolinska Institutet*, Stockholm, Sweden

[b] *Ecobaby Foundation*, The Netherlands

This HENVINET Policy Brief was produced by:

Author:

Arno C Gutleb[a]

Contributors:

Hans Keune[b], Martin Krayer von Krauss[c], Karin E Zimmer[d], Solveig Ravnum[e], Jan L Lyche[d], Erik Ropstad[d], Janneche U Skaare[d,e], Gunnar S Eriksen[e], Janna G Koppe[f], Albertinka J Murk[g], Brooke L Magnanti[h], Alena Bartonova[i], Mike Kobernus[i] and Aileen Yang[i].

[a] Centre de Recherche Public-Gabriel Lippmann, Luxembourg

[b] University of Antwerp, Belgium

[c] WHO Euro, Copenhagen, Denmark

[d] Norwegian School of Veterinary Science

[e] National Veterinary Institute of Norway

[f] EcoBaby Foundation, The Netherlands

[g] Wageningen University, The Netherlands

[h] University Hospitals, Bristol, UK

[i] NILU - Norwegian Institute for Air Research

Contact: gutleb@lippmann.lu

Funding: This project was funded by the EU sixth Framework Programme as part of the HENVINET consortium.

NILU

Universiteit Antwerpen

Centre de Recherche Public Gabriel Lippmann

Veterinærinstituttet National Veterinary Institute

Norges veterinærhøgskole
