# Supplementary material for: Policy relevant results from an expert elicitation on the health risks of phthalates
Source: Environ Health. 2012 Jun 28;11(Suppl 1):S6. doi: 10.1186/1476-069X-11-S1-S6 (PMC3388473; doi:10.1186/1476-069X-11-S1-S6)
Supplement: Additional file 5 - Stakeholder questions — Evaluation questionnaire This questionnaire was used to evaluate the usefulness for the target audience. It was sent to different policy makers and stakeholders. [file 1476-069X-11-S1-S6-S5.pdf]

HENVINET
HEALTH AND ENVIRONMENT NETWORK

| | |
|---|---|
| Do you think the Henvinet approach of expert elicitation process will yield useful information to help decision making (please indicate why)? | |
| Do you find causal diagram easy to understand and a helpful format to inform policy makers and stakeholders about health risk issues and identify priority knowledge gaps for endocrine disrupting compounds? | |
| To what extent and how would you use the expert opinions provided, regarding the extent to which current scientific knowledge justifies a policy intervention elicitation, as a useful contribution to the policy process? | |
| Do you think the procedure and/or format should be improved to be used by policy makers working on endocrine disrupting issues? How could they be made more useful? | |
| Do you have additional advice for us? | |
| Could you propose other experts that you think we should approach for this evaluation? If so, please give us names and contact details. | |
| We will, in addition to the Policy Briefs, write a research article which will be submitted for publication in a scientific journal. All comments given here will be treated anonymously. Still, we will acknowledge everyone who has contributed with full name and affiliation on their wish. Please indicate if you want to be acknowledged anonymously or with name. | |

| | |
|---|---|
| Your name | |
| Your organization | |

**Thank you!**

Please return the questionnaire by April 9th to: solveig.ravnum@vetinst.no
